# Inactivation of the dimeric Rap_pLS20_ anti-repressor of the conjugation operon is mediated by peptide-induced tetramerization

**DOI:** 10.1093/nar/gkaa540

**Published:** 2020-07-13

**Authors:** Isidro Crespo, Nerea Bernardo, Andrés Miguel-Arribas, Praveen K Singh, Juan R Luque-Ortega, Carlos Alfonso, Marc Malfois, Wilfried J J Meijer, Dirk Roeland Boer

**Affiliations:** ALBA Synchrotron Light Source, C. de la Llum 2-26, Cerdanyola del Vallès, 08290 Barcelona, Spain; ALBA Synchrotron Light Source, C. de la Llum 2-26, Cerdanyola del Vallès, 08290 Barcelona, Spain; Centro de Biología Molecular ‘Severo Ochoa’ (CSIC-UAM), C. Nicolás Cabrera 1, Universidad Autónoma, Canto Blanco, 28049 Madrid, Spain; Centro de Biología Molecular ‘Severo Ochoa’ (CSIC-UAM), C. Nicolás Cabrera 1, Universidad Autónoma, Canto Blanco, 28049 Madrid, Spain; Molecular Interactions Facility, Centro de Investigaciones Biológicas Margarita Salas (CSIC), C. Ramiro de Maeztu 9, 28040 Madrid, Spain; Systems Biochemistry of Bacterial Division Lab, Centro de Investigaciones Biológicas Margarita Salas (CSIC), C. Ramiro de Maeztu 9, 28040 Madrid, Spain; ALBA Synchrotron Light Source, C. de la Llum 2-26, Cerdanyola del Vallès, 08290 Barcelona, Spain; Centro de Biología Molecular ‘Severo Ochoa’ (CSIC-UAM), C. Nicolás Cabrera 1, Universidad Autónoma, Canto Blanco, 28049 Madrid, Spain; ALBA Synchrotron Light Source, C. de la Llum 2-26, Cerdanyola del Vallès, 08290 Barcelona, Spain

## Abstract

Quorum sensing allows bacterial cells to communicate through the release of soluble signaling molecules into the surrounding medium. It plays a pivotal role in controlling bacterial conjugation in Gram-positive cells, a process that has tremendous impact on health. Intracellular regulatory proteins of the RRNPP family are common targets of these signaling molecules. The RRNPP family of gene regulators bind signaling molecules at their C-terminal domain (CTD), but have highly divergent functionalities at their N-terminal effector domains (NTD). This divergence is also reflected in the functional states of the proteins, and is highly interesting from an evolutionary perspective. Rap_pLS20_ is an RRNPP encoded on the *Bacillus subtilis* plasmid pLS20. It relieves the gene repression effectuated by Rco_pLS20_ in the absence of the mature pLS20 signaling peptide Phr*_pLS20_. We report here an in-depth structural study of apo and Phr*_pLS20_-bound states of Rap_pLS20_ at various levels of atomic detail. We show that apo-Rap_pLS20_ is dimeric and that Phr*_pLS20_-bound Rap forms NTD-mediated tetramers. In addition, we show that Rap_pLS20_ binds Rco_pLS20_ directly in the absence of Phr*_pLS20_ and that addition of Phr*_pLS20_ releases Rco_pLS20_ from Rap_pLS20_. This allows Rco_pLS20_ to bind the promotor region of crucial conjugation genes blocking their expression.

## INTRODUCTION

Quorum sensing in bacterial cells is a process that allows bacterial cells to exchange information about their state and content (1). The external signals are used by cells to change the expression profile of their genes thereby affecting many processes that are candidates for interference in some way, including the control of expression of virulence factors and the control of bacterial conjugation. In Gram-positive bacteria, the mechanism of action of the signaling molecules is either through activation of a kinase-dependent signaling cascade (the two-component pathway), or by direct interaction with a transcriptional regulator.

The RRNPP family of Gram-positive tetratricopeptide repeat (TPR) proteins (2,3) was so named after discovery of the main representatives of the family, i.e. the proteins Rgg, Rap, NprR, PrgX and PlcR (4–7). In Gram-positive bacteria, RRNPP proteins play a crucial role in quorum sensing (8,9), where they serve as targets of their cognate signaling polypeptide. This peptide is produced, secreted, processed and then reimported into the bacterial cells that also produce the RRNPP. The mature, processed signaling peptide generally consists of a small fragment of the C-terminus of the full-length pre-proprotein, generally about 5–10 amino acids in length (10). Binding of the processed peptide to the C-terminal TPR domain of RRNPP proteins modulates interaction between the RRNPP protein and an effector molecule, leading to further downstream effects. Interaction with effector molecules depends on the N-terminal domain (NTD; effector domain) of the RRNPP (6,11). Thus, the nature of the effector molecule and therefore the function of the RRNPP proteins depend on the type of effector domain incorporated at the N-terminus. The RRNPP-mediated quorum sensing mechanism is involved in the regulation of a variety of bacterial processes including conjugation (e.g. PrgX from the enterococcal plasmid pCF10), sporulation (e.g. RapA from *Bacillus subtilis*) and pathogenicity (e.g. PlcR from *B. cereus*) (7,12), and is found in a range of human commensal or pathogenic Gram-positive bacterial genera such as *Bacillus*, *Streptococcus* and *Enterococcus* (13). Interestingly, RRNPP-like proteins have been found beyond the realm of Gram-positive bacterial genomes, as illustrated by the recent structure determination of a regulator of phage lysis-lysogeny, AimR (14), or in the NlpI protein from Gram-negative *Escherichia coli*, which contains a lipobox motif that anchors it to the membrane (15).

The effector domains of RRNPP proteins known to date can be classified into three groups. The effector domains of the DNA-binding RNPP subclass of proteins are helix-turn-helix (HTH) motifs that are able to negatively regulate protein expression by binding DNA, examples of which are the PrgX protein from *Enterococcus faecalis* and the PlcR protein from *Bacillus thuringiensis*. A second group of proteins, exemplified by *B. subtilis* RapA, RapB, RapE, RapH and RapJ, contains an NTD with phosphatase activity. These proteins form a link in a phosphorylation cascade that translates the peptide signal into a downstream effect. The third group, which includes RapC, RapF, RapG, RapH and RapK, blocks the action of their cognate effector protein by direct interaction, directly or indirectly modulating expression that alters differentiation pathways. Note that RapH belongs to both least two groups as it exhibits both activities. Similarly, NprR contains both a phosphatase and a HTH domain. The variety in functions of different types of RRNPP proteins is also reflected in the differences in oligomerization state and the effect that the peptide can have on oligomerization. Different Rap proteins, even within a single functional class, have been found as monomers, dimers and tetramers, and these oligomerization states may change or remain the same depending on the presence of the peptide. For example, RapJ and RapF are found as monomers (16,17), whereas RapH is reported to be a dimer in solution (18). It seems that the aggregation behavior of RRNPP proteins does not depend directly on the functionality of the NTD, and there is no obvious common theme in how the oligomerization state of the different RRNPP family members relates to the mechanism (19).

*Bacillus subtilis* encodes 11 Rap proteins on its chromosome (6,10,20), and at least five plasmid-encoded variants (21,22). A recently identified *B. subtilis* RRNPP member (22,23), Rap_pLS20,_ is encoded by the Gram-positive *B. subtilis* conjugative plasmid pLS20. It lacks the conserved residues essential for phosphatase activity and has been shown to regulate the conjugation process. The conjugation genes of pLS20 are located in a single large operon that is under the control of the conjugation promoter P_c_. By default, this promoter is repressed by the pLS20-encoded transcriptional regulator Rco_pLS20_ (23). Rap_pLS20_ activates conjugation by acting as an antirepressor of Rco_pLS20_ (22), which induces the formation of a DNA loop by binding to two closely located recognition sequences near the P_c_, thereby inhibiting expression of the conjugation genes and plasmid transfer (23). Rap_pLS20_ is essential to activate transcription of the conjugation genes by releasing Rco_pLS20_-mediated P_c_ repression, which may involve a direct interaction between both. The presence of the Phr*_pLS20_ peptide antagonizes the antirepressive action of Rap_pLS20_, reverting the system to its default state (22). The pre-proprotein Phr_pLS20_ is a 44-amino acid protein, and after secretion, it is subject to a second processing step resulting in the release of the C-terminal 5 amino acids (QKGMY), which can be reimported in an *opp*-dependent manner (22). The genetic switch constituted by the aforementioned components is tightly regulated due to its design. However, the structural basis underlying this mechanism has not been reported.

Here, we demonstrate that Rap_pLS20_ can interact directly with either Rco_pLS20_ or Phr*_pLS20_. In addition, we have determined the crystal structures of Rap_pLS20_ in the apo form as well as in the peptide-bound form to understand the structural mechanism behind peptide-mediated release of antirepression of Rco_pLS20_ by Rap_pLS20_. The structures are validated by SAXS measurements in solution. Surprisingly, our work reveals a tetrameric oligomerization state of Rap_pLS20_ in the presence of the peptide mediated by the NTDs, which has not been observed for other Rap proteins. The oligomerization state and structural changes introduced by peptide binding are compared with information available for other RRNPP members. We find that the peptide-induced change in orientation of the NTDs observed for Rap_pLS20_ is different to that observed for other RRNPP proteins. Thus, the position of the NTDs in apo and peptide-bound forms depends very much on the associated functionality. Implications of the structural findings on the mechanism of action of Rap_pLS20_ are discussed.

## MATERIALS AND METHODS

### Sequence alignments

A set of Rap_pLS20_-like protein sequences was retrieved using an iterative PSI-BLAST search for 11 iterations and standard parameters. The representation of the sequence homology was obtained using the HMM-LOGO program (24).

### Protein expression and purification

Rap_pLS20_ and Rco_pLS20_ were expressed and purified using standard protocols. *Escherichia coli* BL21 (DE3) were transformed with pET-28b carrying the insert of *rap_pLS20_* or *rco_pLS20_* containing a C-terminal His-tag and were inoculated in fresh Luria-Bertani broth (LB) media complemented with 50 μg/ml kanamycin at 37°C overnight. Then, the cells from the overnight culture were collected by centrifugation (4000 × g for 30 min) and suspended in expression media (typically 0.5 l of terrific broth (TB) with 50 μg/ml kanamycin), at a ratio of 30 ml of preculture per liter of medium. Cells in expression media were grown at 37°C until an OD_600_ = 0.8–1 was reached. After that, protein expression was induced overnight at 20°C by addition of 1 mM isopropyl β-d-1-thiogalactopyranoside (IPTG, Omnipur).

After overnight induction, cells were centrifuged at 4000 × g for 30 min, and pellets were frozen and resuspended in lysis buffer at a ratio of 5 ml/g of cells. The lysis buffer used for Rap_pLS20_ contained 50 mM Tris–HCl pH 8.0, 250 mM NaCl, 1% (v/v) glycerol, 10 mM MgCl_2_, 1 mM EDTA, 1 mM β-mercapto-ethanol, 1 mM PMSF, and the buffer used for Rco_pLS20_ contained 20 mM Tris–HCl pH 8.0, 500 mM NaCl, 1% (v/v) glycerol, 10 mM MgCl_2_, 1 mM EDTA, 1 mM β-mercapto-ethanol, 1 mM PMSF. The cell suspension was lysed adding DNAseI to a a final concentration of 200 μg/ml and lysozyme to a final concentration of 100 μg/ml during sonication. Insoluble matter was precipitated by centrifugation (18 000 × g, 30 min), and supernatant was filtered through a 0.22-μm filter and applied to a nickel-charged His-Trap™ HP chelating column 5 ml (GE Healthcare Life Sciences). The column was washed with 10 column volumes of bind buffer (20 mM Tris–HCl pH 8.0, 500 mM NaCl, 5 mM Imidazole) to elute unspecific bound proteins. Bound proteins were eluted using a 5–100 mM imidazole gradient in 20 mM Tris–HCl pH 8.0, 500 mM NaCl. Fractions containing protein were concentrated with an Amicon ultra 10 kDa MWCO (Millipore) and the buffer was exchanged using a PD-10 desalting column (GE Healthcare life sciences) to 20 mM Tris–HCl pH 8.0, 250 mM NaCl, 10 mM MgCl_2_, 1 mM EDTA, 1% (v/v) glycerol buffer for Rap_pLS20_ and to 500 mM NaCl, 20 mM Tris–HCl pH 8.0 buffer for Rco_pLS20_. Typically, a yield of ∼100 mg of Rap_pLS20_ and ∼20 mg of Rco_pLS20_ were obtained from 10 g of pellet. Purity was assessed to be >95% by SDS–PAGE, followed by Coomassie Blue staining. Protein concentration was determined by nanodrop and was used for assays immediately where possible or stored in aliquots at -80°C.

### Analytical size exclusion chromatography on Rap_pLS20_ and Rco_pLS20_ mixtures

To determine the elution volumes of the separate proteins, 25 μg (0.56 nmol) of Rap_pLS20_ and 25 μg (1.2 nmol) Rco_pLS20_, respectively, were injected on a Superdex 200 increase 5/150 GL equilibrated with 500 mM NaCl, 20 mM TRIS pH 8, and eluted at a flow rate of 0.2 ml/min. The concentrations of Rap_pLS20_ and Rco_pLS20_ solutions were 1 mg/ml, or 22.5 μM and 49.4 μM, respectively. For complex binding stoichiometry tests, molar ratios of 1:2, 1:4, 1:1 and 2:1 (Rco_pLS20_:Rap_pLS20_) were prepared and incubated for 30 min on ice before injection. To study the effect of Phr*_pLS20_ peptide on Rap_pLS20_/Rco_pLS20_ complex formation, a 5:1 Phr*_pLS20_:Rap_pLS20_ stoichiometry was used; 25 μl were injected in all cases. All total protein concentrations were within a 0.75–1.25 mg/ml range. To estimate the molecular weights (*M*_w_) of the homo- and heterocomplexes, a calibration of the Superdex 200 increase 5/150 GL column was performed using proteins with known M_w_, which were eluted using the same elution buffer used above. The derived relation between the elution volume (*V*_el_) and *M*_w_ was *V*_el_ = –0.6815 · log(*M*_w_) + 5.1906, with an *R*^2^ = 0.933.

### Sedimentation velocity assays (SV)

Samples of protein Rap_pLS20_ in 20 mM Tris, 250 mM NaCl, 10 mM MgCl_2_, 1 mM EDTA, 0.1 mM β-mercaptoethanol and 1% (v/v) glycerol, pH 7.4, were loaded (320 μl) into 12-mm Epon-charcoal standard double-sector centerpieces. The assays were performed at 48 000 rpm in an XL-I analytical ultracentrifuge (Beckman-Coulter Inc.) equipped with both UV–VIS absorbance and Raleigh interference detection systems, using an An-50Ti rotor. Sedimentation profiles were recorded simultaneously by Raleigh interference and absorbance at 280 nm. Differential sedimentation coefficient distributions were calculated by least-squares boundary modelling of sedimentation velocity data using the continuous distribution *c(s)* Lamm equation model as implemented by SEDFIT (25). These experimental s values were corrected to standard conditions using the program SEDNTERP (26) to get the corresponding standard s values (s20,w). For apo Rap_pLS20_, measurements were performed at 4.5 and 25 μM (0.2–1.1 mg/ml). For the Rap-Phr complex, a Rap_pLS20_ concentration of 4.5 μM (0.2 mg/ml) was used.

### Sedimentation equilibrium assays (SE)

Short column (95 μl) SE experiments of Rap_pLS20_ were carried out at speeds ranging from 7000 to 10 000 rpm and at 280 nm, using the same experimental conditions and instrument as in the SV experiments. A last high-speed run (48 000 rpm) was done to deplete protein from the meniscus region to obtain the corresponding baseline offsets. Weight-average buoyant *M*_w_ of Rap_pLS20_, alone or in the presence of the Phr*_pLS20_ peptide, were obtained by fitting a single-species model to the experimental data using the HeteroAnalysis program (27), once corrected for temperature and solvent composition with the program SEDNTERP (26).

### Fluorescence polarization binding assays

Binding of the Phr*_pLS20_ peptide was assayed in a CLARIOstar plate reader (BMG Labtech) on an OptiPlate-384 Black well plate (PerkinElmer) in 10 μl final assay volume. The Phr*_pLS20_ peptide (GQKGMY, the glycine residue was added at the N-terminus to avoid interference with the dye) used for fluorescence polarization measurements was synthesized with an N-terminal fluorescein label and purified by ThermoFischer Scientific. The buffer used for the fluorescence polarization assays was 20 mM Tris pH 7.5, 150 mM NaCl, 1 mM DTT and 0.01% (v/v) Triton X-100. All buffers were properly degassed under vacuum and oxygen was removed saturating with nitrogen to prevent methionine oxidation. The protein concentration was varied from 0.1–100 μM and the peptide concentration was 50 nM. An excitation wavelength of 485 nm and an emission wavelength of 528 nm were used. For equilibrium competition binding assays, different concentrations of native Phr*_pLS20_ (QKGMY) ranging from 0.6–1250 μM were tested, with constant concentrations of Rap_pLS20_ (10 μM) and 6-FAM- Phr*_pLS20_ (50 nM). The data was measured at 25°C and corrected for background by subtracting the free-labeled peptide background. All the data treatment was done as previously described (28) and the data was fitted to a hyperbolic function for a single binding site using Origin 2018 (OriginLab Corporation).

### SAXS analysis

Different concentrations of Rap_pLS20_, ranging from 0.5 to 5 mg/ml (11.3–113 μM), and Rco_pLS20_, ranging from 1.44 to 14.4 mg/ml for Rco_pLS20_ (70.9–709 μM), were tested to ensure proper scattering and signal detection. In the case of Rap_pLS20_/Phr*_pLS20_ samples, the same concentrations were tested in the presence of 5-fold peptide molar concentration. All the samples were prepared in a final buffer consisting of 15 mM HEPES pH 7.5, 150 mM NaCl, 1 mM DTT. SAXS data on Rap_pLS20_ and Phr*_pLS20_ mixtures were collected at NCD-SWEET beamline (BL11, ALBA Synchrotron, Barcelona). The final buffer was collected for subtraction of Rap_pLS20_ samples, and the buffer plus the highest Phr*_pLS20_ concentration was also collected to ensure that no observable scattering was produced by the Phr*_pLS20_ peptide. Measurements were carried out at 293 K in a quartz capillary of 1.5 mm diameter and 0.01 mm wall thickness. The data (20 frames with an exposure time of 0.5 sec/frame) were recorded using a Pilatus 1M detector (Dectris, Switzerland) at a sample-detector distance of 2.56 m and a wavelength *λ* = 1.0 Å.

Data on mixtures of Rap_pLS20_, Rco_pLS20_ and Phr*_pLS20_ were measured on beamline BM29 at the ESRF, using the automatic sample changer. The concentration ranged from 0.5 to 5 mg/ml (11.3–113 μM) for Rap and 1.44 to 14.4 mg/ml for Rco (70.9–709 μM). Ten consecutive frames were collected with a photon-counting Pilatus 1M detector at a sample-detector distance of 2.85 m, a wavelength *λ* = 0.991 Å and an exposure time of 1 s/frame. A momentum transfer range of 0.036–0.50 Å^−1^ was covered (*q* = 4πsin *θ*/*λ*, *θ* being the scattering angle and *λ* the wavelength of the incident X-ray beam). Data collected during continuous sample flow through the capillary were subtracted from buffer scattering. The frames showing a negligible variation of the radius of gyration (*R*_g_) were merged for further analysis.

*R*_g_ values were obtained from the Guinier approximation *sR*_g_ <1.3 using Primus (29). Distance distribution functions *p*(*r*) and the Porod volumes (*V_ρ_*) were computed from the entire scattering curve using GNOM (30). Buffer subtraction and extrapolation to infinite dilution were performed by using the program package Primus (30) from the ATSAS 2.8.4 software suite. The forward scattering (*I*(0)) and the radius of gyration (*R_g_*) were evaluated by using the Guinier approximation, and the maximum distance *D*_max_ of the particle was also computed from the entire scattering patterns with GNOM. The scattering from the crystallographic models was computed with CRYSOL (31). The volume fractions of the oligomers were determined with OLIGOMER (32), using as probe a set of two structural models corresponding to the monomer and the dimer, and a set of two PDBs corresponding to the dimer and the tetramer. The monomer, dimer and tetramer models were derived from structures presented herein.

### Protein crystallization

Purified Rap_pLS20_ was concentrated to 10 mg/ml in 20 mM Tris–HCl pH 8, 250 mM NaCl, 10 mM MgCl_2_, 1 mM EDTA, 1% (v/v) glycerol. Crystals of Rap_pLS20_ giving the highest resolution were obtained by the sitting-drop vapor-diffusion method at 18°C, by equilibration of drops of 1 μl protein + 1 μl crystallization buffer (10% (w/v) PEG6K, 0.1 M Hepes pH 7, benzamidine hydrochloride 0.1 M) against 100 μl of the crystallization buffer. Needle-shaped crystals were cryo-cooled in liquid nitrogen using a cryo-protecting solution containing reservoir solution supplemented with 20% (v/v) glycerol.

For crystallization experiments of the Rap_pLS20_/Phr*_pLS20_ complex, the peptide was added to the diluted protein at a ratio of 1:5 (Rap_pLS20_/Phr*_pLS20_) and the mixture was concentrated to 10 mg/ml in 20 mM Tris–HCl pH 8, 250 mM NaCl, 10 mM MgCl_2_, 1 mM EDTA, 1% (v/v) glycerol. Crystals of Rap_LS20_- Phr*_pLS20_ were obtained by sitting-drop vapor-diffusion at 18°C, by equilibration of drops of 1 μl protein + 1 μl crystallization buffer (2.5% (w/v) PEG8K, 8% (v/v) ethylene glycol, 0.1 M MES pH 6.8) against 100 μl of the crystallization buffer.

Data collection was performed at ALBA synchrotron Light Source on the BL13-Xaloc beamline (33). The crystals of native Rap_pLS20_ belonged to space group *P*2_1_2_1_2 with two protein molecules in the asymmetric unit. The crystals of the Rap_pLS20_/Phr*_pLS20_ complex belonged to space group C2, with four protein molecules in the asymmetric unit. Data were processed with AutoPROC (Global Phasing Ltd, (34)), using anisotropic resolution cutoffs (35). Data processing statistics are presented in Table 1.

**Table 1.** X-ray data processing and refinement statistics

Data processing statistics	Rap apo (6T3H)	Rap+Phr* (6T47)
Space group	*P* 2_1_ 2_1_ 2	*C* 1 2 1
Unit-cell parameters (Å)	*a* = 111.790, *b* = 174.487, *c* = 49.879	*a* = 116.67, *b* = 93.28, *c* = 167.69
Unit-cell angles (°)	α = β = γ = 90	α = γ = 90, β = 94.97
Resolution range (Å)^a^	87.2–3.04 (3.09–3.04)	45.4–2.450 (2.492–2.450)
No. of unique reflections	18645 (797)	63446 (3200)
Spherical completeness (%)	95.3 (82.5)	96.1 (96.8)
Ellipsoidal completeness (%)	95.3 (82.5)	95.9 (95.6)
Redundancy	4.6 (4.5)	2.2 (2.2)
Mean *I*/σ(*I*)	14.8 (1.4)	10 (0.3)
*R*_meas_ (%)^b^	7.5 (104.1)	4.4 (154.7)
Refinement statistics		
*R*_work_^c^ (%)	20.13	19.93
*R*_free_^d^ (%)	25.93	25.68
Ramachandran		
Favored (%)	92.95	96.4
Disallowed (%)	1.13	1.31
R.M.S.D.		
Bond lengths (Å)	0.004	0.01
Bond angles (°)	0.734	1.503
Chirality		
Mean *B* value (Å^2^)	95.4	73.3

^a^Numbers in parentheses represent values in the highest resolution shell.

^b^ *R*_meas_ = ∑*hkl* [*N*/*N* – 1]^1/2^∑_i_ |*Ii*(*hkl*) – <*I*(*hkl*)>|/∑_*hkl*_∑_*i*_*Ii*(*hkl*) where *N* is the multiplicity of a given reflection, *Ii*(*hkl*) is the integrated intensity of a given reflection and <*I*(*hkl*)> is the mean intensity of multiple corresponding symmetry-related reflections.

^c^ *R*_work_ = ∑ ||*F*_obs_| – |*F*_calc_||/∑ |*F*_obs_|, where |*F*_obs_| and |*F*_calc_| are the observed and calculated structure factor amplitudes, respectively.

^d^ *R*_free_ is the same as *R*_work_ but calculated with a 5% subset of all reflections that was never used in refinement.

### Structure refinement

The TPR domain of the structure of the RapF protein with PDB code 4I9E (11) was used for molecular replacement by the program phaser (36). The native model of Rap_pLS20_ was then used for molecular replacement for the Rap_pLS20_/Phr*_pLS20_ data. Crystallographic refinement of the models was done using phenix 1.12–2829 (37) and manual building in Coot (38), using the 2*F*_o_ − *F*_c_ and *F*_o_ − *F*_c_ electron-density maps from refinement. Refinement statistics are presented in Table 1. Figures were prepared using PyMOL (The PyMOL Molecular Graphics System, Version 1.8 Schrödinger, LLC). Sequence and secondary structure were visualized using ESPript (39). The apo structure of Rap_pLS20_ was deposited as PDB code 6T3H and the peptide-bound structure was deposited as PDB code 6T46.

### Superposition of Rap_pLS20_ monomers

To quantify changes in conformation between different, yet equivalent structures, the displacements of the equivalent atoms were expressed as a root-mean-square deviation (RMSD) of the atomic positions after superpositioning different parts of the structures. The main chain atoms of either the matching full length sequence (residues 9–361), or of residues 80-361 (CTD), or of residues 9–68 (NTD), respectively, of each of the crystallographically independent monomers of the Rap_pLS20_ apo and Rap_pLS20_/Phr*_pLS20_ structures were superposed using the least squares algorithm implemented in Coot. The superposition of the main chain atoms of residues 268–361, corresponding to residues in H13-H17, was calculated using the *fit* command in Pymol. The *rms_cur* command in Pymol was used to calculate the RMSD of the main chain atoms of residues 9–68 after superposition of residues 80–361.

## RESULTS

This study shows that the oligomerization state of Rap_pLS20_ is concentration dependent. For the purpose of comparison, Supplementary Table S1 lists the concentrations used for the solution experiments.

### Overall structure of Rap_pLS20_

**Figure 1.**
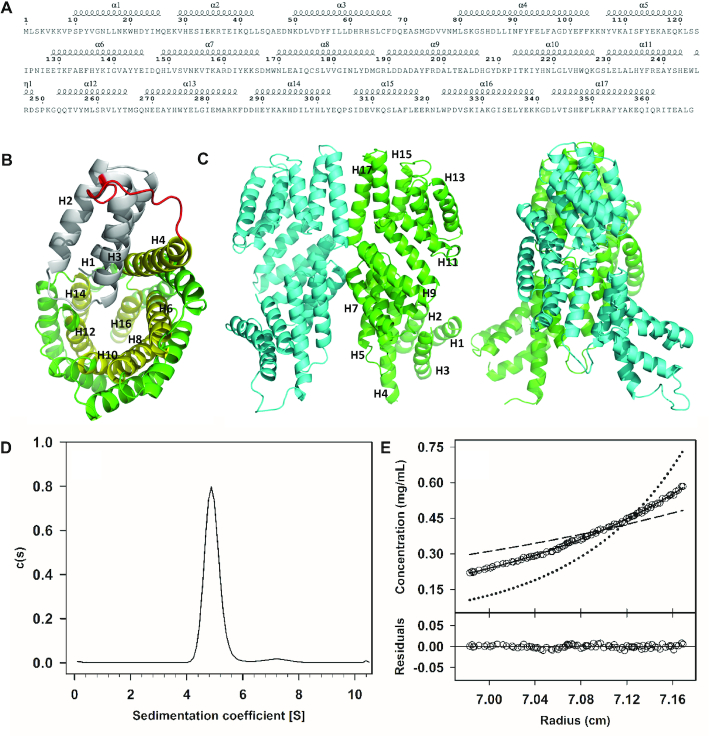
Overall crystallographic structure and ultracentrifuge results of apo Rap_pLS20_. (**A**) Sequence and secondary structure of the apo structure. (**B**) N-terminal view of a cartoon representation of the apo Rap_pLS20_ structure. Helices H1–H3 of the N-terminal domain are shown in gray, the H3–H4 loop in red and the even-numbered TPR helices lining the solenoid in yellow. Uneven-numbered helices are shown in green. (**C**) Cartoon representation of the side view of the dimeric structure of apo Rap_pLS20_ in two orientations. (**D**) Sedimentation velocity assay showing the sedimentation coefficient distribution *c(s)* corresponding to 4.5 μM (0.20 mg/ml) purified Rap_pLS20_. (**E**) Sedimentation equilibrium assay. Upper part: concentration gradient of experimental data for Rap_pLS20_ (empty circles) are presented together with best-fit analysis assuming protein monomer (dashed line), dimer (black line), or tetramer (dotted line) species. Lower part: Difference between experimental data and estimated values for a protein dimer model (residuals).

The apo structure shows that Rap_pLS20_ is all α-helical, consisting of 17 antiparallel helices that are connected by short loops (Figure 1A and B). The C-terminal region consists of 14 helices forming 7 bi-helical TPR motifs, making up the typical solenoid structure associated with TPR folds (2). The solenoid covers more than a full turn, resulting in an interaction of the C-terminus with H5 and H6. The channel of the solenoid structure is open towards the N-terminus, but tightens towards the C-terminus, mainly due to the presence of the sidechains of residues R261 and W225.

The inner surface of the solenoid is lined by the even-numbered helices from H4 to H16 (Figure 1B), which form the pocket to which the peptide binds. The TPR topology is lost at the N-terminal end of helix H4, as helix H3 is located towards the inner lining and helix H2 towards the outer lining of the solenoid, an inversion with respect to helices H4 and H5. The loop between helices H3 and H4, indicated in red in Figure 1B, therefore marks the boundary between the NTD and the C-terminal TPR domain. This loop is the longest loop in the structure, consisting of 13 residues, and shows up poorly in the electron density maps, indicating a high degree of flexibility. Helices 1–3 form an antiparallel three-helix bundle, where helices H1 and H3 interact with the N-terminal region of helix H4. Helix H2 packs with helix H1 and the opposing side of helix H3 is exposed to the exterior part of the protein.

The three helices of the NTD are approximately parallel and therefore do not form a HTH topology. Instead, their configuration resembles that of the TPR fold, but the domain is tilted with respect to the C-terminal TPR domain. The linker between helices 3 and 4 mentioned above provides the flexibility required for this change in orientation. This implies flexibility of the functionally important NTD domain, which relays peptide binding to a downstream response (6).

Rap_pLS20_ forms dimers in the packing of the crystal structure in a similar fashion to the *B. subtilis* phosphatase RapH (18). The interaction involves the protein surface of helices H5–H7 and H16–H17, including the C-terminal residues (Figure 1B and C). The N-terminal entrances of the channels of the solenoid structures face outwards with respect to the dimer interface. The buried solvent-accessible surface of the dimer interface in the apo structure is presented in Table 2. It is the largest buried surface area in the structure, and corresponds to the interface having the strongest interactions present in the structures according to strength indicators (see Table 2).

**Table 2.** Relevant parameters indicating the strengths of the interface areas between the C-terminal TPR dimerization domains (CTD) and N-terminal tetramerization domains (NTD), respectively, for the different monomers in the asymmetric units in apo and peptide-bound Rap_pLS20_ structures

Structure	Interactions	Area (Å^2^)	Δ*G*	Δ*G*/*P*	CSS
Rap Apo	CTD of Chain A with chain B	1432	−10.2	0.341	0.79
Rap Pep	CTD of Chain A with chain C	1606	−12.2	0.285	0.453
	CTD of Chain E with chain G	1440	−13.6	0.205	1.00
Rap Apo tetramer	NTDs of Chain A with chain B*	1347	−24.1	0.01	0.85
Rap Pep	NTDs of Chain G with chain E*	1207	−16.6	0.05	1.00
	NTDs of Chain C with chain A*	1306	−22.1	0.01	0.516

*The interaction is with the indicated chain of a symmetry-related molecule.

### Rap_pLS20_ oligomerization state in solution

To determine whether the apo form of Rap_pLS20_ also forms dimers in solution as observed in the crystal structure, we carried out two complementary analytical ultracentrifugation (AU) approaches: sedimentation velocity (SV) and sedimentation equilibration (SE) experiments. In SV experiments, more than 96% of Rap_pLS20_ was observed as a single species with an experimental sedimentation coefficient of 4.9 S at 4.5 μM (0.20 mg/ml). This sedimentation coefficient, after correction to standard conditions (S20,w = 5.4S), was compatible with the theoretical mass of a slightly elongated Rap_pLS20_ dimer (*f*/*f*_0_ = 1.3) (Figure 1D). Since the apparent *M*_w_ obtained in SV analyses is influenced by the shape of the protein complex, we also performed SE experiments at concentrations ranging from 4.5 to 25 μM or 0.20 to 1.1 mg/ml (Figure 1E). The buoyant mass obtained was 22 930 ± 110 Da, corresponding to a molar mass of 89 970 ± 440 Da, using a partial specific volume of 0.7363, confirming that Rap_pLS20_ (theoretical *M*_w_ of 44.4 kDa) behaves predominantly as a dimer in solution.

### Structural comparison and evolutionary link

Given the low sequence homology among members of the RRNPP family (6), a comparison of the structure of Rap_pLS20_ with other known structures in the PDB database could be very insightful (40). The full sequence of the apo structure was subjected to a PDB eFold search (41), summarized in Supplementary Table S2, in which the RRNPP proteins are grouped according to their function and source organism. The table is complemented with relevant RRNPP and non-RRNPP entries that were not identified by the eFold search. The search resulted in the retrieval of most but not all RRNPP proteins, indicating that there are significant structural differences between Rap_pLS20_ and some RRNPP members, particularly in the NTD. Most interestingly, a number of non-related TPR proteins were found to have a greater structural similarity to Rap_pLS20_ than some of the RRNPP proteins, suggesting that the RRNPP proteins and the non-RRNPP hits stem from a common ancestor. When investigating the structural similarities, it became clear that the evolutionary link between these proteins lies in the peptide binding function of the TPR domain. For example, human LGN is one of the proteins that were found to have a TPR domain that is structurally similar to RRNPP proteins. Human LGN is involved in mitotic spindle orientation of eukaryotic cell division, and in order to do so, it must be able to interact with different partner proteins (42,43) by binding to a polypeptide segment of each. However, the peptides to which LGN binds are generally much longer than the processed signaling molecules in bacterial quorum sensing, and RRNPP proteins are unable to bind long peptides because the peptide binding site at the C-terminus forms a cul-de-sac in which only short peptides are able to fit. A second functionally and structurally important adaptation between RRNPP and LGN is related to the NTD, which is absent in LGN.

### Fluorescence polarization binding assay

**Figure 2.**
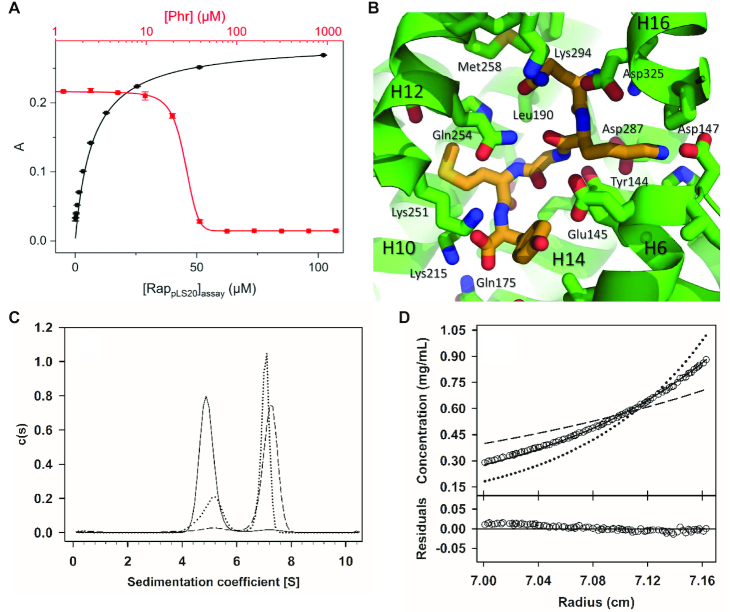
Interaction of the Phr*_pLS20_ peptide with Rap_pLS20_. (**A**) Rap_pLS20_/Phr*_pLS20_ binding affinity as determined by fluorescence polarization. The black-colored data points represent the fluorescence polarization of the 6-FAM-labeled Phr*_pLS20_ peptide as a function of Rap_pLS20_ concentration. The red-colored data points shows the results of a fluorescence anisotropy competition assay with unlabeled Phr*_pLS20_. The black and red lines represent the fit of the experimental data to the Hill equation for a single binding site. Error bars represent standard deviations from four samples. (**B**) Stick representation of the crystal structure of the peptide-bound crystallographic structure, showing the carbon atoms of the peptide in orange and those of Rap_pLS20_ in green. Interacting residues of Rap_pLS20_ are indicated. (**C**) Sedimentation coefficient distributions, *c*(*s*), obtained from SV assays at 280 nm with 4.5 μM of apo Rap_pLS20_, showing the shift in the *s*-value of this protein after addition of Phr* at 4.5 μM (dotted line) or 15 μM (dashed trace) relative to Rap_pLS20_ alone (solid trace). (**D**) Sedimentation equilibrium assay. Upper part: concentration gradient of experimental data for Rap_pLS20_ with Phr*_pLS20_ at 15 μM (empty circles) are presented together with best-fit analysis assuming protein dimer (dashed line), tetramer (black line) or hexamer (dotted line) species. Lower part: Difference between experimental data and estimated values for the protein tetramer model (residuals).

We have determined by fluorescence anisotropy the affinity of a synthetic Phr*_pLS20_ peptide to Rap_pLS20_ using a competition assay between a chromophore-labelled (6-FAM-Phr*_pLS20_) and an unlabeled Phr*_pLS20_ peptide following a previously described protocol (44). Briefly, fluorescence anisotropy was measured for samples containing fixed concentrations of Rap_pLS20_ and 6-FAM-Phr*_pLS20_ and increasing concentrations of native Phr*_pLS20_. The binding data obtained in these fluorescence polarization assays were fitted (Figure 2A) to the Hill-Langmuir equation (45), resulting in a Hill coefficient of 1 (no cooperativity in the interaction). The determined IC_50_ was 26.73 ± 0.27 μM, which corresponds to a *K*_D_ value of 7.42 μM, which is in the same range as the 3.1 μM *K*_D_ value calculated for the RapF-PhrF* pair (11), but significantly less than the 0.03 μM calculated for NprR and its cognate peptide NprX (46). The difference in peptide affinity between Rap proteins and NprR may be explained by the difference in length of the peptide (five amino acids for the Phr peptides and eight for NprX). The *K*_D_ value allowed us to estimate the proper ratio of Rap_pLS20_/Phr*_pLS20_ required to obtain high percentage of protein bound to the peptide. Unless stated otherwise, a ratio of 1:5 (Rap_pLS20_:Phr*_pLS20_) was chosen for the experiments described herein, as under these conditions a 98.8% Phr*_pLS20_ was calculated to be bound to the protein.

### Structures of peptide-bound Rap_pLS20_

Having resolved the structure of the apo form, we were interested in revealing the structural effects of Phr*_pLS20_ binding on Rap_pLS20_. Crystals diffracting at 2.45 Å were obtained, and X-ray analyses revealed that the peptide-bound Rap_pLS20_ crystallized in a different space group with distinct cell parameters (see Table 1), consisting of four independent chains in the asymmetric unit. The electron density of the peptide was clearly observed in all chains (Supplementary Figure S1). The approximate location and orientation of the peptide in the TPR domain of Rap_pLS20_ is preserved compared with other members of the RRNPP family (not shown). The peptide is oriented along the solenoid axis of the TPR domain, with its N-terminus bound in a closed pocket pointing towards the C-terminus of Rap_pLS20_. The C-terminus of the peptide, however, points towards an open channel, which provides clues about how the peptide enters the active site. Interestingly, superpositions with other RRNPP-peptide structures show that small relative displacements of the peptide occur along the solenoid direction (not shown). Recognition of the peptide by Rap_pLS20_ involves residues from helices H6, H8, H10, H12, H14 and H16 (Figure 2B).

The sequence of the mature Phr*_pLS20_ peptide corresponds to the last five amino acids of the pre-proprotein, residues forty to forty-four, with the sequence QKGMY. Phr*_pLS20_ adopts an extended conformation, with residues Q40 and K41 pointing in opposite directions with respect to the main chain, as do residues M43 and Y44 (Figure 2B). Thus, residues K41 and Y44 point towards helix H6, whereas Q40 and M43 point towards the interface between helices H10 and H12. K41 interacts with residues at the top of helix H6. This part of H6 interacts in turn with helices H16 and H17, which are involved in the dimer interface. The peptides of the four monomers of the peptide-bound Rap_pLS20_ structure were superposed to visualize the movement of Rap_pLS20_ structural elements with respect to the peptide. This analysis shows that in one of the Phr*_pLS20_-bound chains, the C-terminal helices H13-H17 of the TPR moves downwards by 2.6 Å, bringing these helices close to the helices H5–H7.

The superpositions of the peptides revealed a significant degree of freedom of movement, which was further analyzed by superposing the structural elements of the protein. For this, the different chains of both the apo Rap_pLS20_ and Rap_pLS20_ peptide-bound structures were superposed (Supplementary Tables S3 and S4). To get insight into the structural changes induced in Rap_pLS20_ due to peptide binding, we calculated the average distance existing between the main chain atoms of the apo- Rap_pLS20_ structure to the equivalent main chain atoms of the superposed Phr*_pLS20_–Rap_pLS20_ structure, and expressed these as RMSDs. The superposition of all possible residues showed that the overall structure did not change much, evidenced by the fact that the RMSDs ranged from 1.33 to 3.54 Å (Supplementary Table S3). However, visual inspection of these superpositions suggested that the orientation of the NTDs changed relative to the CTDs. We therefore superposed only the CTDs of the apo and peptide-bound monomers (RMSDs of 0.58–2.86 Å) and the NTDs of all monomers (RMSDs of 0.64–1.50 Å) (see Supplementary Table S3). In addition, we calculated the RMSDs of the NTDs after superposition of the CTDs, which were in the range of 1.30 to 7.0 Å (Supplementary Table S4). The significant increase in the RMSDs of the NTDs after superposition of the CTDs compared to those of the superposition of the NTDs confirmed the change in relative orientation of the domains. We found that in the presence of the peptide, the NTDs move in a single direction and have a tendency to swing outward, away from the solenoid axis of the TPR. This movement can be observed to various degrees and was most pronounced for two of the four monomers (chains C and G) in the Phr*_pLS20_–Rap_pLS20_ structure, which each belong to the two different respective dimers.

**Figure 3.**
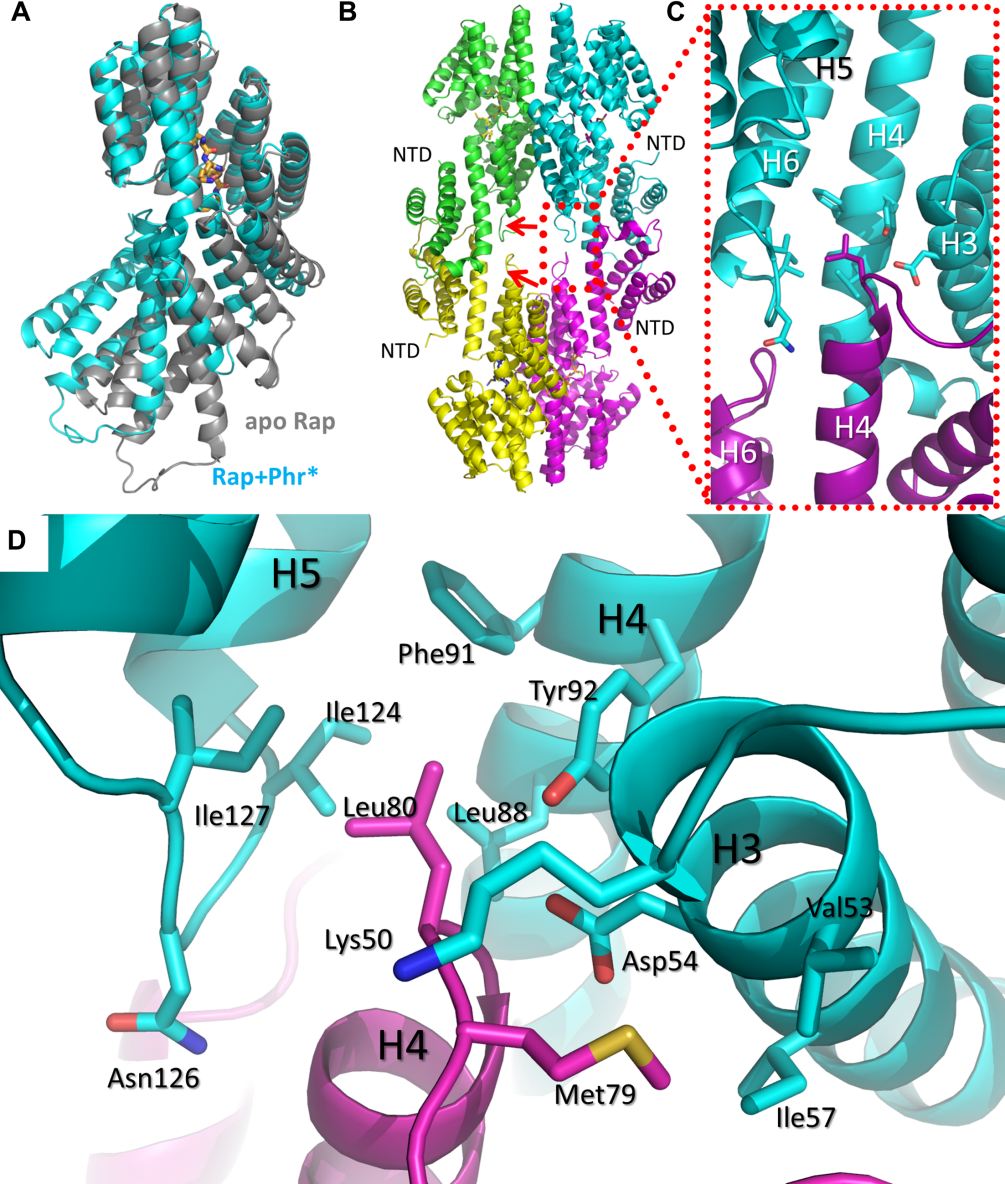
Rap_pLS20_ tetramerization. (**A**) Cartoon representation of the superposition of the C-terminal domains of one of the peptide-bound chains (cyan) on one of the apo chains (gray), highlighting The shift in the N-terminal domain (NTD). The peptide carbon atoms are shown in orange. (**B**) Side view of the peptide-bound tetramer. The red arrows indicate the loops connecting helices H4 and H5. (**C**) Zoom of the area around the N-terminus of helix H4, showing the insertion of this helix into the opposite monomer. (**D**) Close up view of the hydrophobic pocket of M79 and L80 in the formation of the tetramer.

To delineate further which regions were relatively rigid or flexible, superpositions of subsets of the helices of the CTDs were performed and inspected visually. These analyses showed that the C-terminal helices H13–H17 were invariant when compared to the preceding structural elements, as evidenced by RMSDs of 0.380–0.740 Å after the superposition of helices H13-H17 (residues 268–361) of all monomers of the apo and peptide-bound structures (Supplementary Table S3). The superposition revealed that helices H6 and H7 (residues 129–166) of the TPR domain move inwards towards the center of the solenoid where the peptide binds, and upwards towards the C-terminus. The extent of this movement is distinct for the four chains of the Phr*_pLS20_-bound Rap_pLS20_ and ranges from 2.1 to 3.5 Å. This ample range of movement confirms that the TPR domain retains a high level of flexibility in the presence of the bound peptide. The movements in helices H6 and H7 are related to an outward movement of the N-terminal 3-helix bundle. In fact, the extent of both movements is correlated: the N-terminal bundles swing out over a larger distance when the TPR rearrangement is more pronounced. The correlation between the displacement of helices H6 and H7 and the tendency of the NTDs to move outward is likely functionally important for the response of Rap to the peptide. Figure 3A shows the positions of the effector domain with respect to the TPR domain for one monomer of the apo structure (chain B) and one monomer of the peptide-bound structure (chain G).

### Effect of Phr*_pLS20_ on Rap_pLS20_ oligomerization in solution

To investigate possible effects of peptide binding on the oligomerization state of Rap_pLS20_ in solution, we performed SV and SE ultracentrifugation experiments of Rap_pLS20_ in the presence of synthetic Phr*_pLS20_. Interestingly, the presence of Phr*_pLS20_ induced a change in oligomerization state of Rap_pLS20_ in a dose-dependent manner, resulting in the formation of Rap_pLS20_ tetramers. At a 1:1 stoichiometry, there was still a small trace of Rap_pLS20_ dimers, which was no longer observed when the peptide was present in excess with a 3:1 stoichiometry. Thus, an equilibrium between the dimer and tetramer was observed, as was the case for apo Rap_pLS20_. In fact, in the absence of the peptide, a small but measurable concentration-dependent presence of tetramers was observed in SV experiments of apo Rap_pLS20_, which increased from 1.1% at 4.5 μM (0.20 mg/ml) to 3.6% at 25 μM (1.1 mg/ml).

SV assays showed a shift of the sedimentation coefficient of Rap_pLS20_ from 4.9 S, corresponding to the protein dimer, to an s-value of 7.1 S, compatible with a slightly elongated shape of the Rap_pLS20_ tetramer (*f*/*f*_0_ = 1.44) (Figure 2C). To confirm the oligomerization state of Rap_pLS20_ in the presence of the peptide, regardless of the hydrodynamic shape, we carried out a SE experiment of Rap_pLS20_ at different concentrations (0–15 μM or 0–0.67 mg/ml) in the presence of a 1:3 stoichiometry (Rap_pLS20_:Phr*_pLS20_). SE showed a buoyant mass of 44 300 Da, corresponding to a molar mass of 174 000 ± 740 Da, in good agreement with the expected *M*_w_ for the protein tetramer (Figure 2D). Together, these data show that Rap_pLS20_ forms tetramers in solution in the presence of Phr*_pLS20_.

### The tetramerization interface of Rap_pLS20_

We next checked if tetramerization of Rap_pLS20_ observed by AUC is also reflected in the crystal structures. When analyzing the intermolecular contacts in the peptide-bound crystal structure, we found an additional interface that explains the tetrameric configuration of Rap_pLS20_, which surprisingly was also found in the structure of the apo form. This second interface formed through interactions between the helices 3 of the NTDs of the interacting molecules, and by insertion of the N-terminus of helix H4 between helices H4-6 and the N-terminus of helix H3 of the opposite molecule. We will refer to this interaction as the **foot-2-foot interaction** (Figure 3B and C). Of the many interactions found in the interface, we highlight here M79 and L80 located in H4 (Figure 3D), which forms part of the CTD and interact substantially with residues in the H4–H5 loop of the opposing monomer. Within the PSI-BLAST (47) set of proteins, which includes Rap_pLS20_-like proteins, we found that L80 is highly conserved (I, L or V in homologous proteins) and sits in a hydrophobic pocket lined with residues F91, Y92, L121, P125, I127, K131 of a chain from the opposite dimer. Of these interacting residues, F91-Y92 are 100% identical among the BLAST sequences, and 121 is mainly L with a smaller percentage of I. In addition, P125 and I127 are very well conserved (mainly P, then A/E/D/T for P125 and 100% for I127). K131 is Q in most sequences, followed by K, then smaller populations of Y, H, R. Interestingly, within the 11 Rap proteins of *B. subtilis*, the conservation of the residues forming this pocket is much smaller. Except for F91-Y92 and L121, the remaining pocket residues and L80 and the preceding loop, containing M79, are poorly conserved. This may indicate that peptide-induced tetramerization is common among Rap_pLS20_-like proteins, but does not occur among the genomic *B. subtilis* Rap proteins characterized so far.

Table 2 gives the interface strengths for the tetramer interfaces in the apo structure (one crystallographically independent interface) and the peptide-bound structure (two crystallographically independent interfaces). The strength of the interface area of the foot-2-foot interactions shows that the N-terminal interactions are viable interactions that are stronger than other contacts in the crystal lattice.

### SAXS analysis of the particle size of Rap_pLS20_ and Rco_pLS20_, with and without Phr*_pLS20_

We have established the formation of Rap_pLS20_ tetramers using AUC and we have derived a possible interface of this interaction from the crystal structures. The contacts observed in the crystal surface could be crystallographic artefacts, though, that may not occur in solution. Therefore, this interface required confirmation, using different techniques to provide additional insights into the oligomerization behavior and shape of Rap_pLS20_ in solution in the presence and absence of the Phr*_pLS20_ peptide. Multiple size exclusion chromatography (SEC) and SAXS analyses were performed on (combinations of) Rap_pLS20_, Rco_pLS20_ and Phr*_pLS20._

We analyzed apo Rap_pLS20_ at concentrations of 11 μM to 0.11 mM (0.5–5 mg/ml) and Rco_pLS20_ at concentrations of 70.9–709 μM (1.44–14.4 mg/ml), both in the absence and presence of Phr*_pLS20_ at a 5× molar excess. A summary of the most important global SAXS parameters is given in Table 3.

**Table 3.** SAXS data obtained for samples of Rap_pLS20_, Rco_pLS20_, or mixed samples in the absence and presence of Phr*_pLS20_. The average value obtained from curves at different concentrations are given, the values in brackets correspond to the highest and lowest values determined for the different concentrations

Sample	Phr*_LS20_	*R*_g_ (nm)	*V*_porod_ (nm^3^)	*R*_max_ (nm)
Rap_pLS20_	−	3.8 (3.44–4.03)	195 (162–217.5)	14.6 (14.3–14.9)
	+	4.5 (4.21–4.52)	231 (174.1–263.5)	14.8 (14.0–16.0)
Rco_pLS20_	−	(4.52–6.19)	(206–443)	(15.8–21.7)
Rap_pLS20_/Rco_pLS20_	−	(5.3–11.9)	(294–1281)	(18.5–60.4)
	+	(4.35–4.86)	(255–286)	(15.0–17.0)

Comparison of SAXS data obtained for Rap_pLS20_ showed that the presence of Phr*_pLS20_ provoked an increase in the volume and weight of Rap_pLS20_ particles (Table 3). The presence of the peptide resulted in the following specific increases: the radius of gyration increased from 3.8 to 4.5 nm; the Porod volume increased from 195 to 231 nm^3^; and the maximum distance derived from the *P(r)* changed from 14.6 to 14.8 nm. These data are consistent with a tetrameric Rap_pLS20_/Phr*_pLS20_ oligomerization as observed by AUC data and the X-ray structures.

The size indicators obtained for Rco_pLS20_ at a concentration of approximately 1.44 mg/ml (70.9 μM), were similar to those obtained for Rap_pLS20_ (Table 3). This indicates that Rco_pLS20_ (theoretical *M*_w_ of 20.3 kDa) has a similar effective size as Rap_pLS20_ (theoretical *M*_w_ = 44.4 kDa) in solution, consistent with AUC data published previously showing that Rco_pLS20_ is a tetramer in solution (23), and the AUC presented herein showing that Rap_pLS20_ behaves as a dimer. At higher concentrations, however, the parameters indicate that the effective size of the particles increase, suggesting that Rco_pLS20_ forms complexes of higher *M*_w_ at increasing concentrations.

### SEC and SAXS analysis show that the Rap_pLS20_-Rco_pLS20_ complex is disrupted by Phr*_pLS20_

Since we were also interested in studying the interaction between Rap_pLS20_ and Rco_pLS20_ and the effect of the signaling peptide on this possible interaction, we measured scattering curves of mixtures of Rap_pLS20_ and Rco_pLS20_ at different stoichiometries and concentrations, in the absence and presence of Phr*_pLS20_ (Table 3). In the absence of signaling peptide, mixtures of Rap_pLS20_ and Rco_pLS20_ exhibited a several-fold increase in all particle size indicators, independent of their relative stoichiometries. This shows that a complex between Rap_pLS20_ and Rco_pLS20_ is indeed formed, confirming direct interaction between these proteins. When mixtures of Rap_pLS20_, Rco_pLS20_ and Phr*_pLS20_ were analyzed by SAXS, the overall indicators of particles size and shape (Table 3) were similar to those of the Rco_pLS20_ protein alone and the Rap_pLS20_/Phr*_pLS20_ complex, regardless of the relative concentrations of the components.

Next, we analyzed the oligomerization behavior of Rap_pLS20_ and Rco_pLS20_ using analytical size exclusion chromatography (SEC) at a total protein concentration ranging between 0.75 and 1.25 mg/ml and estimated the M_w_ of the different complexes based on a calibration using proteins of known *M*_w_ (Supplementary Table S5). The results show that Rap_pLS20_ and Rco_pLS20_ alone eluted at similar volumes, with estimated weights of 101 and 94.4 kDa, respectively, corresponding to Rap_pLS20_ dimers and Rco_pLS20_ tetramers. The formation of apo Rap_pLS20_ dimers in solution is in agreement with AUC data reported above and with previously reported SEC results (48). The tetrameric form of Rco_pLS20_ is in agreement with AUC results reported before (23), but does not agree with previously published SEC and sucrose gradient results (48).

As mentioned above, higher order complexes were observed by SAXS for mixtures of Rap_pLS20_ and Rco_pLS20_ in absence of the signaling peptide. SEC experiments of mixtures of Rap_pLS20_ and Rco_pLS20_ showed similar results (Supplementary Figure S2C–F). Addition of Phr*_pLS20_ to the Rap_pLS20_ and Rco_pLS20_ mixtures disrupted these higher order complexes in SEC (Supplementary Figure S2H), in agreement with SAXS results. Together, the SAXS and SEC data are consistent with Phr*_pLS20_-dependent disruption of Rco_pLS20_–Rap_pLS20_ complexes and concomitant formation of a Rap_pLS20_ tetramer.

### SAXS confirms the formation of apo Rap_pLS20_ dimers and Rap_pLS20_/Phr*_pLS20_ foot-2-foot tetramers in solution

**Figure 4.**
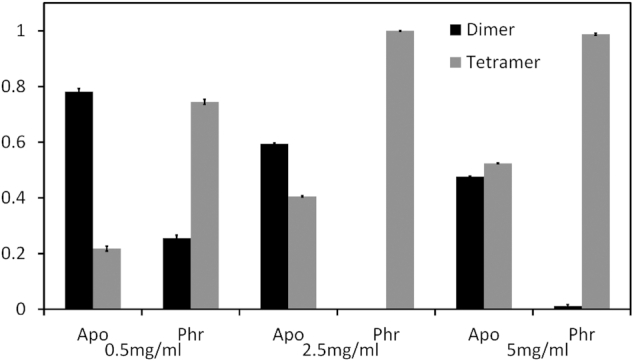
SAXS determined concentration-dependent dimer:tetramer stoichiometry of the apo and peptide-bound form of Rap_pLS20_. The vertical bars represent the proportion of dimers and foot-2-foot tetramers of Rap_pLS20_ and Rap_pLS20_/Phr*_pLS20_ samples at three different concentrations, as calculated with OLIGOMER (32).

In order to assess the agreement between X-ray structure and SAXS data, models of the monomer, dimer and tetramer from the crystal structures were used to calculate a fit with the SAXS scattering curves (Supplementary Table S6). Based on the crystal lattices, two different tetramer models were generated: one representing a foot-to-foot induced tetramer, and the second generated using (weaker) side-to-side CTD interactions found in the crystal structures. SAXS data of Rap_pLS20_ showed that at all tested concentrations, the best model to explain the curves was always a combination of dimer (Figure 1C) and foot-2-foot tetramer (Figure 3B). Unacceptable fits were invariably obtained for combinations of the Rap_pLS20_ monomer, the dimer and for the CTD-based, side-to-side tetramer (Supplementary Figure S3 and Supplementary Table S4). The best fit for the apo Rap_pLS20_ SAXS curve at 0.5 mg/ml (11.3 μM) was obtained with a dimer:tetramer ratio of 0.78:0.22. The percentage of foot-2-foot tetramers increased at higher Rap_pLS20_ concentrations; e.g. at 5 mg/ml (0.11 mM) the Rap_pLS20_ dimer:tetramer ratio was 0.48:0.52 (Figure 4 and Supplementary Table S6). Thus foot-2-foot tetramers are found to coexist with dimers in solutions of apo Rap_pLS20_, and their proportion depended on the Rap_pLS20_ concentration.

Importantly, the presence of Phr*_pLS20_ caused a considerable increase in the percentage of Rap_pLS20_ tetramers, which was particularly noticeable at low concentrations of Rap_pLS20_ (Figure 4). For example, at 0.5 mg/ml (11.3 μM) the presence of the signaling peptide caused the percentage of tetramers to increase from 22 to 75%. It is also worth noting that, as was the case for apo Rap_pLS20_, the proportion of tetramers was augmented at increasing peptide-bound Rap_pLS20_ concentrations (maintaining the stoichiometries between Rap_pLS20_ and Phr*_pLS20_ constant). In fact, 100% of Rap_pLS20_ was in tetramer form at Rap_pLS20_ concentrations above 2.5 mg/ml (57.7 μM). In summary, the SAXS data and the results of the ultracentrifugation experiments described above show that, in the absence of the peptide, Rap_pLS20_ has a tendency to form foot-2-foot tetramers at high concentrations. However, formation of these tetramers is strongly enhanced by the presence of the signaling peptide (Figure 4).

## DISCUSSION

### Oligomerization state of Rap_pLS20_

In a previous study, we demonstrated that Rap_pLS20_ activates expression of conjugation genes by relieving Rco_pLS20_-mediated repression of the main conjugation promoter P*_c_*, and that this anti-repression activity of Rap_pLS20_ is inhibited by Phr*_pLS20_ (22). The results of the different experimental approaches applied in these studies show that Rap_pLS20_ forms dimers which have an intrinsic ability to produce tetramers, but that tetramer formation is particularly stimulated by the cognate mature signaling peptide Phr*_pLS20_. These results strongly suggest that tetramerization is a crucial feature for inactivation of Rap_pLS20_. In the crystal structure, we observed two possible modes by which Rap_pLS20_ may tetramerize. The solution experiments allowed the identification of the functional configuration of the Rap_pLS20_ tetramerization, which we named the foot-2-foot interaction. Interestingly, in this complex, the dimer-dimer interface involves mainly interactions between the NTDs. This proposed model of the tetramer is also compatible with AUC data, since the frictional ratio indicates a slightly elongated model for the dimer and an even more elongated model for the tetramer. The deviations in the *M*_w_ estimations from SEC data may indicate non-globular molecular shapes and therefore also agree with the dimer and foot-2-foot models proposed by us.

The foot-2-foot interfaces seem to be stronger in the apo crystal structure compared to the peptide-bound structure (see Table 2). There are several possible explanations for this observation. First, the resolution of the apo structure is higher and the electron density of the loop region is clearer, allowing a better positioning of the interacting residues. Second, the lower resolution of the peptide-bound structure may obscure bridging water molecules and ions, which would increase the strength of the interaction surface. And third, crystal contacts may push the NTDs to a non-preferred orientation in the apo structure that favors tetramer formation. However, our combined data based on results from different techniques provide compelling evidence that Phr*_pLS20_ binding enhances Rap_pLS20_ homotetramerization via a foot-2-foot interaction. Notably, AUC and SAXS experiments showed that Rap_pLS20_ foot-2-foot tetramers can also form in solution in the absence of the signaling peptide, and that the proportion of the tetramers is concentration dependent. Our data therefore suggest that Rap_pLS20_ dimers and tetramers are in a concentration-dependent equilibrium that is shifted in favor of the tetramer upon peptide binding.

### Interaction between Rap_pLS20_ and Rco_pLS20_ in presence and absence of Phr*_pLS20_

Our SAXS results firmly establish that Rap_pLS20_ and Rco_pLS20_ interact to form larger complexes, as indicated by the increase in particle size in solution. Interestingly, Rap_pLS20_/Rco_pLS20_ mixtures additionally containing Phr*_pLS20_ yielded particle size parameters in the same range as those obtained for the Rap_pLS20_/Phr*_pLS20_ complex and Rco_pLS20_ alone, indicating that Phr*_pLS20_ interferes with interactions between Rap_pLS20_ and Rco_pLS20_. These data were corroborated by SEC, and overall show that Phr*_pLS20_ restores the repressive action of Rco_pLS20_ (22,23) by modulating the direct interaction between Rap_pLS20_ and Rco_pLS20_.

By extrapolating the fact that the NTD of RRNPP proteins determines their functionality, Rap_pLS20_ and Rco_pLS20_ interact most likely through the NTD of Rap_pLS20_. There is a similarity to the RapF/ComA system, in that ComA and Rco_pLS20_ are both DNA binding proteins that harbor an HTH domain. RapF binds ComA through a surface that mimics the surface of DNA (16). This interaction is mediated partly through a 27-residue linker that connects the NTD to the CTD (16). However, the corresponding linker in Rap_pLS20_ is 14 residues shorter, and the electrostatic surface of Rap_pLS20_ does not show pronounced negative charges. These observations indicate that the NTD of Rap_pLS20_ does not likely mimic DNA, suggesting that Rap_pLS20_ binds to its modulator protein Rco_pLS20_ in a very different way. A possible implication of this is that Rap_pLS20_ may not interact with the Rco_pLS20_ N-terminal DNA-binding domain, but rather with its CTD, which resembles the lambda-phage C1 repressor (Interpro IPR010982 family). Future structural studies will be performed to determine how Rco_pLS20_ interacts with Rap_pLS20_.

### Complex formation of RRNPP proteins compared with Rap_pLS20_

Dimerization like that observed here in Rap_pLS20_ has been observed in the apo form of RRNPP proteins that bind DNA such as PlcR and PrgX, but is uncommon for other Rap proteins. Most Rap proteins have been reported to be monomers in solution, including RapF, RapH and RapK (16), and RapJ (17). RapH, however, does form dimers (18). For RapF (16) and RapJ (17), large conformational changes in the N-terminus have been observed in peptide binding, but no related changes in oligomerization state have been reported. In these Rap proteins, a large internal conformational change was proposed to account for the functional effects of peptide binding. In contrast, a change in oligomerization state was found for PlcR (4) and PrgX (49,50) upon peptide binding. The effect of peptide binding was, however, distinct for the two proteins: PlcR tetramerized upon peptide binding, whereas the two different peptides that compete for PrgX binding have stabilizing and destabilizing effects on tetramerization, respectively. Interestingly, the SAXS results of PlcR showed an extended volume, indicating formation of higher order oligomerization. At low protein concentrations, these volumes resemble to a great extent the foot-2-foot tetramers presented herein and were interpreted as tetramers, albeit through interactions in the N-terminal part of the TPR domain (4).

In Rap_pLS20_, binding of the peptide results in changes of several alpha helices of the TPR domain, which in turn translates into changes in orientation of the NTDs such that they allow foot-2-foot interactions between opposing NTDs. In the peptide-bound structure, two crystallographically independent tetramer configurations were found, which are formed through foot-2-foot interactions between two symmetry-related dimers. In each of the tetramers, one of the NTDs moves outwards, thereby modifying the interaction interface. In the tetramer formed by chains G and E and their symmetry-related counterparts, the displacement of the NTD of chain G is most pronounced. In the tetramer formed by chains A and C and their symmetry-related counterparts, displacement of the NTD of chain C is most pronounced, but this displacement is smaller than that of chain G. The combined effect in the G/E tetramer is a stronger interaction than that observed for the A/C tetramer, judged by the strength of the buried surface area (Table 2). Curiously, the strength of the dimer interface in both peptide-bound tetramers is slightly decreased, suggesting that peptide weakens the dimer interface (Table 2).

The movement of the NTD with respect to its CTD observed here for Rap _pLS20_ upon Phr*_pLS20_ peptide binding has also been observed for other members of the Rap family, such as RapF (PDB code 4i9c), RapJ (4gyo) and NprR (5dbk). Intriguingly, the movement of the NTD of Rap_LS20_ is opposite to that observed in these other structures. In fact, RapJ (17) and RapF (11) contract upon peptide binding, causing the N-terminal bundle to merge with the TPR domain. The position of the NTDs in these structures is not compatible with the tetrameric structures observed for Rap_pLS20_. The fact that a tetrameric oligomerization state is not observed for RapJ and RapF is consistent with this observation.

Tetramers of RRNPP proteins have been observed using a range of techniques, but atomic models of tetramers have been proposed only for NprR and PrgX. In PrgX, tetramerization has been proposed to occur through the CTDs of the apo protein (51). PrgX tetramers are destabilized upon binding of the cognate cCF10 peptide, so this mechanism is very distinct from the mechanism proposed here for Rap_pLS20_. For NprR, however, the NTDs are important for the formation of tetramers, and the dimer-to-tetramer transition is induced by the peptide (46). The peptide-dependent transformation from dimer to tetramer of NprR is reminiscent of the behavior of Rap_pLS20_. The interactions in the NprR tetramer occur between the TPR and NTD domains, though foot-2-foot interactions like those observed for Rap_pLS20_ were not found for the peptide-bound structure of NprR. Instead, the dimers are rotated 90° with respect to each other, and the NTDs bind on the sides of the TPR domains. Interestingly, the NTDs in the peptide-bound NprR structure move inward as is the case for the monomeric RapF and RapJ; however, this does not lead to tetramers for these Rap proteins.

The foot-2-foot tetramerization observed exclusively for Rap_pLS20_ may be the consequence of the very different NTD of this protein. Interestingly, interactions between the helices immediately following the NTDs are a common theme between the tetramerization interactions of Rap_pLS20_ and of the other types of interactions described above for other members of the RRNPP family, suggesting that this part of the TPR domain is of direct importance in translating peptide binding into a functional effect.

### Implications of the mechanism of Rap_pLS20_

The homotetramerization caused by the foot-2-foot interactions of the NTDs of Rap_pLS20_ provides an explanation for the activation of the Rco_pLS20_ partner (Figure 3). In the absence of Phr*_pLS20_, the NTDs are positioned such that they allow the interaction with Rco_pLS20_. However, upon binding the signaling peptide, the NTDs shift outwards, facilitating the formation of the homotetramer, leading to a change of the interaction surface of the NTDs that is no longer available for interactions with Rco_pLS20_. This change leads to a release and subsequent reactivation of Rco_pLS20_, which is again able to bind to the P_c_ (22). Thus, the structural changes introduced in the Rap_pLS20_ structure determine whether the conjugation process will be activated or not.

In summary, our approach combining four different techniques has been crucial in the elucidation of the oligomerization behavior of Rap_pLS20_ and its interaction partners. We have determined the X-ray structures of apo Rap_pLS20_ and the Phr*_pLS20_-bound structure. These structures demonstrated that binding of the peptide changes the position of the NTDs with respect to the CTDs and result in a transition of homodimers to homotetramers of Rap_pLS20_, through a foot-2-foot interaction between the repositioned NTDs. SAXS, SEC and AUC experiments confirm this model, which differs substantially from the model proposed for other RRNPP family members. The differences in peptide-dependent oligomerization behavior seem to be closely related to the function of the NTDs of Rap proteins. Thus, the main (and possibly only) function of the C-terminal TPR domain seems to be in binding the peptide, whereas the NTD is responsible for exerting the biological function of the protein in response to binding of the cognate peptide.

## DATA AVAILABILITY

Atomic coordinates and structure factors for the Rap_pLS20_ and the Rap_pLS20_ peptide-bound crystal structures have been deposited in the Protein Data bank under accession numbers 6T3H and 6T46, respectively.

## Supplementary Material

gkaa540_Supplemental_File

## Notes

Present address: Praveen K. Singh, Max Planck Institute for Terrestrial Microbiology, Karl-von-Frisch-Straße 10, Marburg 35043, Germany.

## References

[1] Rutherford S.T., Bassler B.L. Bacterial quorum sensing: its role in virulence and possibilities for its control. Cold Spring Harb. Perspect. Med. 2012; 2:a012427. 23125205 10.1101/cshperspect.a012427 PMC3543102

[2] D’Andrea L.D., Regan L. TPR proteins: the versatile helix. Trends Biochem. Sci. 2003; 28:655–662. 14659697 10.1016/j.tibs.2003.10.007

[3] Lamb J.R., Tugendreich S., Hieter P. Tetratrico peptide repeat interactions: to TPR or not to TPR. Trends Biochem. Sci. 1995; 20:257–259. 7667876 10.1016/s0968-0004(00)89037-4

[4] Declerck N., Bouillaut L., Chaix D., Rugani N., Slamti L., Hoh F., Lereclus D., Arold S.T. Structure of PlcR: Insights into virulence regulation and evolution of quorum sensing in Gram-positive bacteria. Proc. Natl. Acad. Sci. U.S.A. 2007; 104:18490–18495. 17998541 10.1073/pnas.0704501104 PMC2141804

[5] Dunny G.M., Berntsson R.P.-A. Enterococcal sex pheromones: evolutionary pathways to complex, two-signal systems. J. Bacteriol. 2016; 198:1556–1562. 27021562 10.1128/JB.00128-16 PMC4959283

[6] Neiditch M.B., Capodagli G.C., Prehna G., Federle M.J. Genetic and structural analyses of RRNPP intercellular peptide signaling of Gram-Positive bacteria. Annu. Rev. Genet. 2017; 51:311–333. 28876981 10.1146/annurev-genet-120116-023507 PMC6588834

[7] Rocha-Estrada J., Aceves-Diez A.E., Guarneros G., de la Torre M. The RNPP family of quorum-sensing proteins in Gram-positive bacteria. Appl. Microbiol. Biotechnol. 2010; 87:913–923. 20502894 10.1007/s00253-010-2651-y

[8] Dunny G.M., Leonard B.A. Cell-cell communication in Gram-positive bacteria. Annu. Rev. Microbiol. 1997; 51:527–564. 9343359 10.1146/annurev.micro.51.1.527

[9] Bareia T., Pollak S., Eldar A. Self-sensing in Bacillus subtilis quorum-sensing systems. Nat. Microbiol. 2018; 3:83–89. 29038467 10.1038/s41564-017-0044-z PMC5739288

[10] Pottathil M., Lazazzera B.A. The extracellular Phr peptide-Rap phosphatase signaling circuit of Bacillus subtilis. Front. Biosci. 2003; 8:d32–d45. 12456319 10.2741/913

[11] Gallego del Sol F., Marina A. Structural basis of Rap phosphatase inhibition by Phr peptides. PLoS Biol. 2013; 11:e1001511. 23526880 10.1371/journal.pbio.1001511 PMC3601957

[12] Kohler V., Keller W., Grohmann E. Regulation of Gram-positive conjugation. Front. Microbiol. 2019; 10:1134. 31191478 10.3389/fmicb.2019.01134 PMC6540685

[13] Perez-Pascual D., Monnet V., Gardan R. Bacterial cell-cell communication in the host via RRNPP peptide-binding regulators. Front. Microbiol. 2016; 7:706. 27242728 10.3389/fmicb.2016.00706 PMC4873490

[14] Gallego Del Sol F., Penades J.R., Marina A. Deciphering the molecular mechanism underpinning phage arbitrium communication systems. Mol. Cell. 2019; 74:59–72. 30745087 10.1016/j.molcel.2019.01.025 PMC6458997

[15] Ohara M., Wu H.C., Sankaran K., Rick P.D. Identification and characterization of a new lipoprotein, NlpI, in Escherichia coli K-12. J. Bacteriol. 1999; 181:4318–4325. 10400590 10.1128/jb.181.14.4318-4325.1999 PMC93934

[16] Baker M.D., Neiditch M.B. Structural basis of response regulator inhibition by a bacterial anti-activator protein. PLoS Biol. 2011; 9:e1001226. 22215984 10.1371/journal.pbio.1001226 PMC3246441

[17] Parashar V., Jeffrey P.D., Neiditch M.B. Conformational change-induced repeat domain expansion regulates Rap phosphatase quorum-sensing signal receptors. PLoS Biol. 2013; 11:e1001512. 23526881 10.1371/journal.pbio.1001512 PMC3601965

[18] Parashar V., Mirouze N., Dubnau D.A., Neiditch M.B. Structural basis of response regulator dephosphorylation by Rap phosphatases. PLoS Biol. 2011; 9:e1000589. 21346797 10.1371/journal.pbio.1000589 PMC3035606

[19] Do H., Kumaraswami M. Structural mechanisms of peptide recognition and allosteric modulation of gene regulation by the RRNPP family of quorum-sensing regulators. J. Mol. Biol. 2016; 428:2793–2804. 27283781 10.1016/j.jmb.2016.05.026 PMC4938729

[20] Perego M., Hanstein C., Welsh K.M., Djavakhishvili T., Glaser P., Hoch J.A. Multiple protein-aspartate phosphatases provide a mechanism for the integration of diverse signals in the control of development in B. subtilis. Cell. 1994; 79:1047–1055. 8001132 10.1016/0092-8674(94)90035-3

[21] Meijer W.J.J., Wisman G.B.A., Terpstra P., Thorsted P.B., Thomas C.M., Holsappel S., Venema G., Bron S. Rolling-circle plasmids from Bacillus subtilis: complete nucleotide sequences and analyses of genes of pTA1015, pTA1040, pTA1050 and pTA1060, and comparisons with related plasmids from Gram-positive bacteria. FEMS Microbiol. Rev. 1998; 21:337–368. 9532747 10.1111/j.1574-6976.1998.tb00357.x

[22] Singh P.K., Ramachandran G., Ramos-Ruiz R., Peiró-Pastor R., Abia D., Wu L.J., Meijer W.J.J. Mobility of the native Bacillus subtilis conjugative plasmid pLS20 is regulated by intercellular signaling. PLoS Genet. 2013; 9:e1003892. 24204305 10.1371/journal.pgen.1003892 PMC3814332

[23] Ramachandran G., Singh P.K., Luque-Ortega J.R., Yuste L., Alfonso C., Rojo F., Wu L.J., Meijer W.J.J. A complex genetic switch involving overlapping divergent promoters and DNA looping regulates expression of conjugation genes of a gram-positive plasmid. PLoS Genet. 2014; 10:e1004733. 25340403 10.1371/journal.pgen.1004733 PMC4207663

[24] Schuster-Böckler B., Schultz J., Rahmann S. HMM logos for visualization of protein families. BMC Bioinformatics. 2004; 5:7. 14736340 10.1186/1471-2105-5-7 PMC341448

[25] Schuck P. Size-distribution analysis of macromolecules by sedimentation velocity ultracentrifugation and lamm equation modeling. Biophys. J. 2000; 78:1606–1619. 10692345 10.1016/S0006-3495(00)76713-0 PMC1300758

[26] Laue T.M., Shah B.D., Ridgeway T.M., Pelletier S.L. Harding S.E., Rowe A.J., Horton J.C. Computer-Aided Interpretation of analytical sedimentation data for proteins. Analytical Ultracentrifugation in Biochemistry and Polymer Science. 1992; Cambridge Royal Society of Chemistry 90–125.

[27] Cole J.L. Analysis of heterogeneous interactions. Methods Enzymol. 2004; 384:212–232. 15081689 10.1016/S0076-6879(04)84013-8 PMC2924680

[28] Rossi A.M., Taylor C.W. Analysis of protein-ligand interactions by fluorescence polarization. Nat. Protoc. 2011; 6:365–387. 21372817 10.1038/nprot.2011.305 PMC3160472

[29] Petoukhov M.V., Franke D., Shkumatov A.V., Tria G., Kikhney A.G., Gajda M., Gorba C., Mertens H.D.T., Konarev P.V., Svergun D.I. New developments in the ATSAS program package for small-angle scattering data analysis. J. Appl. Crystallogr. 2012; 45:342–350. 25484842 10.1107/S0021889812007662 PMC4233345

[30] Franke D., Petoukhov M.V., Konarev P.V., Panjkovich A., Tuukkanen A., Mertens H.D.T., Kikhney A.G., Hajizadeh N.R., Franklin J.M., Jeffries C.M. et al. ATSAS 2.8: a comprehensive data analysis suite for small-angle scattering from macromolecular solutions. J. Appl. Crystallogr. 2017; 50:1212–1225. 28808438 10.1107/S1600576717007786 PMC5541357

[31] Svergun D., Barberato C., Koch M.H.J. *CRYSOL* – a program to evaluate X-ray solution scattering of biological macromolecules from atomic coordinates. J. Appl. Crystallogr. 1995; 28:768–773.

[32] Konarev P.V., Volkov V.V., Sokolova A.V., Koch M.H.J., Svergun D.I. PRIMUS: a Windows PC-based system for small-angle scattering data analysis. J. Appl. Crystallogr. 2003; 36:1277–1282.

[33] Juanhuix J., Gil-Ortiz F., Cuní G., Colldelram C., Nicolás J., Lidón J., Boter E., Ruget C., Ferrer S., Benach J. Developments in optics and performance at BL13-XALOC, the macromolecular crystallography beamline at the alba synchrotron. J. Synchrotron Radiat. 2014; 21:679–689. 24971961 10.1107/S160057751400825X PMC4073956

[34] Vonrhein C., Flensburg C., Keller P., Sharff A., Smart O., Paciorek W., Womack T., Bricogne G. Data processing and analysis with the *autoPROC* toolbox. Acta Crystallogr. Sect. D. 2011; 67:293–302. 21460447 10.1107/S0907444911007773 PMC3069744

[35] Tickle I.J., Flensburg C., Keller P., Paciorek W., Sharff A., Vonrhein C., Bricogne G. 2018; STARANISO.

[36] McCoy A.J., Grosse-Kunstleve R.W., Adams P.D., Winn M.D., Storoni L.C., Read R.J. *Phaser* crystallographic software. J. Appl. Crystallogr. 2007; 40:658–674. 19461840 10.1107/S0021889807021206 PMC2483472

[37] Adams P.D., Afonine P.V., Bunkóczi G., Chen V.B., Davis I.W., Echols N., Headd J.J., Hung L.-W., Kapral G.J., Grosse-Kunstleve R.W. et al. *PHENIX*: a comprehensive Python-based system for macromolecular structure solution. Acta Crystallogr. Sect. D. 2010; 66:213–221. 20124702 10.1107/S0907444909052925 PMC2815670

[38] Emsley P., Lohkamp B., Scott W.G., Cowtan K. Features and Development of Coot. Acta Crystallogr. D - Biol. Crystallogr. 2010; 66:486. 20383002 10.1107/S0907444910007493 PMC2852313

[39] Robert X., Gouet P. Deciphering key features in protein structures with the new ENDscript server. Nucleic Acids Res. 2014; 42:W320–W324. 24753421 10.1093/nar/gku316 PMC4086106

[40] Berman H.M., Westbrook J., Feng Z., Gilliland G., Bhat T.N., Weissig H., Shindyalov I.N., Bourne P.E. The Protein Data Bank. Nucleic Acids Res. 2000; 28:235–242. 10592235 10.1093/nar/28.1.235 PMC102472

[41] Krissinel E., Henrick K. Inference of macromolecular assemblies from crystalline state. J. Mol. Biol. 2007; 372:774–797. 17681537 10.1016/j.jmb.2007.05.022

[42] Takayanagi H., Yuzawa S., Sumimoto H. Structural basis for the recognition of the scaffold protein Frmpd4/Preso1 by the TPR domain of the adaptor protein LGN. Acta Crystallogr. F, Struct. Biol. Commun. 2015; 71:175–183. 25664792 10.1107/S2053230X14028143 PMC4321472

[43] Zhu J., Wen W., Zheng Z., Shang Y., Wei Z., Xiao Z., Pan Z., Du Q., Wang W., Zhang M. LGN/mInsc and LGN/NuMA complex structures suggest distinct functions in asymmetric cell division for the Par3/mInsc/LGN and Gαi/LGN/NuMA pathways. Mol. Cell. 2011; 43:418–431. 21816348 10.1016/j.molcel.2011.07.011 PMC3158460

[44] Rossi A.M., Taylor C.W. Analysis of protein-ligand interactions by fluorescence polarization. Nat. Protoc. 2011; 6:365–387. 21372817 10.1038/nprot.2011.305 PMC3160472

[45] Gesztelyi R., Zsuga J., Kemeny-Beke A., Varga B., Juhasz B., Tosaki A. The Hill equation and the origin of quantitative pharmacology. Arch. Hist. Exact Sci. 2012; 66:427–438.

[46] Zouhir S., Perchat S., Nicaise M., Perez J., Guimaraes B., Lereclus D., Nessler S. Peptide-binding dependent conformational changes regulate the transcriptional activity of the quorum-sensor NprR. Nucleic Acids Res. 2013; 41:7920–7933. 23793817 10.1093/nar/gkt546 PMC3763537

[47] Altschul S.F., Madden T.L., Schäffer A.A., Zhang J., Zhang Z., Miller W., Lipman D.J. Gapped BLAST and PSI-BLAST: a new generation of protein database search programs. Nucleic Acids Res. 1997; 25:3389–3402. 9254694 10.1093/nar/25.17.3389 PMC146917

[48] Rosch T.C., Graumann P.L. Induction of plasmid conjugation in bacillus subtilis is bistable and driven by a direct interaction of a Rap/Phr quorum-sensing system with a master repressor. J. Biol. Chem. 2015; 290:20221–20232. 26112413 10.1074/jbc.M115.664110 PMC4536431

[49] Bae T., Dunny G.M. Dominant-negative mutants of prgX: evidence for a role for PrgX dimerization in negative regulation of pheromone-inducible conjugation. Mol. Microbiol. 2001; 39:1307–1320. 11251846 10.1111/j.1365-2958.2001.02319.x

[50] Kozlowicz B.K., Shi K., Gu Z.-Y., Ohlendorf D.H., Earhart C.A., Dunny G.M. Molecular basis for control of conjugation by bacterial pheromone and inhibitor peptides. Mol. Microbiol. 2006; 62:958–969. 17038121 10.1111/j.1365-2958.2006.05434.x PMC2655123

[51] Shi K., Brown C.K., Gu Z.-Y., Kozlowicz B.K., Dunny G.M., Ohlendorf D.H., Earhart C.A. Structure of peptide sex pheromone receptor PrgX and PrgX/pheromone complexes and regulation of conjugation in Enterococcus faecalis. Proc. Natl. Acad. Sci. U.S.A. 2005; 102:18596–18601. 16339309 10.1073/pnas.0506163102 PMC1317922

