# Supplementary material for: Inactivation of the dimeric RappLS20 anti-repressor of the conjugation operon is mediated by peptide-induced tetramerization
Source: Nucleic Acids Res. 2020 Jul 13;48(14):8113–27. doi: 10.1093/nar/gkaa540 (PMC7430634; doi:10.1093/nar/gkaa540)
Supplement: gkaa540_Supplemental_File [file gkaa540_supplemental_file.pdf]

Supplementary material

Suppl. Table 1. Protein concentrations ranges of Rap_{pLS20} used in the solution experiments.

Technique	$[Rap_{pLS20}]$	
	µM	mg/mL
AUC	4.5 – 25	0.20 – 1.11
SEC	16.9 – 28.1	0.75 – 1.25
SAXS	11.3 – 113	0.5 – 5

Suppl. Table 2. Relevant similar structures of Rap_{pLS20}, based on an eFold search of Rap_{pLS20} with similar structures manually added.

**Protein**	**Source**	**N-ter /Function**	**PDB code : Chain**	**Oligo**	**Components/ Remarks**	**Lowest eFold Z score (RMSD)**	**Ref**
RappLS20	*Bacillus subtilis*	PPI	6T3H	Dimer			This work
	E9RIY6			Dimer	Phr (QKGMY)		This work
RapF	Bacillus subtilis	PPI (Reported monomer)	4I9E	Dimer (no foot2foot)		12 (2.0)	(1)
	P71002		4I9C	Dimer (no foot2foot)	QRGMI	8.70 (2.5)	(1)
			3ULQ	Dimer (no foot2foot)	+ComA DBD	10.3 (2.4)	(2) (RapF mimics DNA) AUC: monomer
RapI	*B subtilis* 168 (P96649)	Phosphatase	4I1A	Dimer (no foot2foot)		11.0 (2.0)	(3)
RapH	*B subtilis* 168	Phosphatase	3Q15	Dimer	Spo0F	8.17 (2.9)	(4)

	(Q59HN8)	and PPI		(no prev oligo data, SEC monomer)<br>No foot2foot			AUC: dimer
RapJ	*Bacillus subtilis* O34327	Phosphatase (Reported monomer)	4GYO	Dimer (Reported monomer)<br>No foot2foot	PhrC (ERGMT)	9.74 (2.0)	(3)<br>(ref 28: monomer)
NprR	*Bacillus cereus* (G5DDY8)	Phosphatase and HTH-DBD	4GPK (no HTH)	Dimer<br>Tetramerization, resembles foot2foot	NprX (SSKPDIVG)	5.12 (3.0)	(5)<br>AUC:
NprR	*Bacillus thuringiensis* (M1Q3P9)	Phosphatase and HTH-DBD	5DBK (no HTH)	Dimer<br>No foot2foot	-	7.92 (3.0)	(6)
PlcR	*Bacillus thuringiensis* (Q3EZ40)	HTH	2QFC	Dimer (Nter crossover)<br>No foot2foot	PapR (LPFEF)	Not listed	(7): SAXS: Dimers, tetramers with peptide
		HTH	3U3W	Dimer (Nter crossover)<br>No foot2foot	DNA	Not listed	(8)
PrgX	*Enterococcus faecalis*	HTH	2AW6 (ΔCT)	Dimer (Nter crossover), tetramer in structure through TPR Cter	cCF10(pep)	Not listed	(9): no info on oligo
	Q04114		2AWI (Y153C)	Dimer (Nter crossover), tetramer in structure through TPR Cter		Not listed	(9): no info on oligo
			2AXU	Dimer (Nter crossover), tetramer in structure through TPR Cter		Not listed	(9): no info on oligo
			2AXV (Y153C)			Not listed	(9): no info on oligo
			2AXZ	Dimer (Nter	cCF10(pep)	Not listed	(9): no info

				crossover), tetramer in structure through TPR Cter			on oligo
			2GRL (ΔCT)	Dimer (Nter crossover)	iCF10	Not listed	(10)
			2GRM (Y231C)	Dimer (Nter crossover)	iCF10	Not listed	(10)
NlpI	*E. coli* P0AFB1	Lipobox motif	1XNF	Dimer (Nter transverses structure)		6.33 (2.80)	(11)
			5WQL			6.33 (2.87)	(12)
Rgg	*Streptococcus dysgalactiae*	HTH	4YV6	Dimer (Nter crossover)		Not listed	(13)
	A0A0J9X288	HTH	4YV9	Dimer (Nter crossover)	Cyclosporine A	Not listed	(13)
		HTH	5W4M	Dimer (Nter crossover)	C45S mutation	Not listed	(14)
		HTH	5W4N	Dimer (Nter crossover)	C45S mutation	Not listed	(14)
Non NPRR							
LGN	Human P81274	Cell adhesion/ Signalling	4WND:A			8.54 (2.23)	(15)
		Cell adhesion/ Signalling	5A6C:A			7.82 (2.4)	(16)
		Cell adhesion/ Signalling	3SF4:C			5.90 (2.7)	(17)
		Cell adhesion/ Signalling	4WNF:A			2.94 (3.8)	(15)
		Cell adhesion/ Signalling	4WNE:A			5.11 (3.4)	(15)
		Cell adhesion/ Signalling	4WNG:A			5.17 (3.4)	(15)
LGN	Human & mouse Q8VDU0	Signalling	4G2V:A			7.84 (2.5)	(18)
			3RO2:A			7.78 (2.5)	(19)
LGN	*Drosophila melanogaster* Q9VB22	Cell Cycle	5A7D:D			5.71 (2.4)	TBP
CDC26	Human Q8NHZ8	CC ligase	3HYM:D			7.07 (2.4)	(20)
MalT	*E. coli* K12 P06993	Transcription activator	1HZ4:A			4.80 (3.2)	(21)
Parmer	*Parabacteroi*	Unknown	4R7S:A			5.24 (3.3)	JCSG

	des merdae ATCC 43184 A7AK45	(JCSG)					
Coat.e	*Bos Taurus* Q28104	Transport	3MKR:A			2.63 (3.9)	(22)
Coat.e	*Mus musculus/S. cerevisiae* P11076	Transport	5A1Y:Z			3.07 (3.9)	(23)
			5A1V:Z			3.07 (3.9)	(23)
PEX5	Human P50542	Transport	2C0M:A			2.35 (3.6)	(24)
			2J9Q:A			2.04 (3.6)	(25)
TbPEX5	*T. brucei brucei* Q57W55	Transport	3CVP:A			1.94 (4.0)	(26)
			3CV0:A			1.91 (4.0)	(26)
TbPEX5	*T. brucei brucei* Q9U7C3	Transport	3CVQ:A			2.00 (3.9)	(26)
			3CVL:A			2.02 (4.0)	(26)

References to Table S2

1. Gallego del Sol, F. and Marina, A. (2013) Structural basis of Rap phosphatase inhibition by Phr peptides. *PLoS Biol.*, **11**, e1001511.

2. Baker, M.D. and Neiditch, M.B. (2011) Structural basis of response regulator inhibition by a bacterial anti-activator protein. *PLoS Biol.*, **9**, e1001226.

3. Parashar, V., Jeffrey, P.D. and Neiditch, M.B. (2013) Conformational change-induced repeat domain expansion regulates Rap phosphatase quorum-sensing signal receptors. *PLoS Biol.*, **11**, e1001512.

4. Parashar, V., Mirouze, N., Dubnau, D.A. and Neiditch, M.B. (2011) Structural basis of response regulator dephosphorylation by Rap phosphatases. *PLoS Biol.*, **9**, e1000589.

5. Zouhir, S., Perchat, S., Nicaise, M., Perez, J., Guimaraes, B., Lereclus, D. and Nessler, S. (2013) Peptide-binding dependent conformational changes regulate the transcriptional activity of the quorum-sensor NprR. *Nucleic Acids Res.*, **41**, 7920–7933.

6. Perchat, S., Talagas, A., Poncet, S., Lazar, N., Li de la Sierra-Gallay, I., Gohar, M., Lereclus, D. and Nessler, S. (2016) How Quorum Sensing Connects Sporulation to Necrotrophism in Bacillus thuringiensis. *PLoS Pathog.*, **12**, 1–21.

7. Declerck, N., Bouillaut, L., Chaix, D., Rugani, N., Slamti, L., Hoh, F., Lereclus, D. and Arold, S.T. (2007) Structure of PlcR: Insights into virulence regulation and evolution of quorum sensing in Gram-positive bacteria. *Proc. Natl. Acad. Sci. U. S. A.*, **104**, 18490–18495.

8. Grenha, R., Slamti, L., Nicaise, M., Refes, Y., Lereclus, D. and Nessler, S. (2013) Structural basis for the

activation mechanism of the PlcR virulence regulator by the quorum-sensing signal peptide PapR. *Proc. Natl. Acad. Sci. U. S. A.*, **110**, 1047–1052.

9. Shi, K., Brown, C.K., Gu, Z.-Y., Kozlowicz, B.K., Dunny, G.M., Ohlendorf, D.H. and Earhart, C.A. (2005) Structure of peptide sex pheromone receptor PrgX and PrgX/pheromone complexes and regulation of conjugation in Enterococcus faecalis. *Proc. Natl. Acad. Sci. U. S. A.*, **102**, 18596–18601.

10. Kozlowicz, B.K., Shi, K., Gu, Z.-Y., Ohlendorf, D.H., Earhart, C.A. and Dunny, G.M. (2006) Molecular basis for control of conjugation by bacterial pheromone and inhibitor peptides. *Mol. Microbiol.*, **62**, 958–969.

11. Wilson, C.G.M., Kajander, T. and Regan, L. (2005) The crystal structure of NlpI: A prokaryotic tetratricopeptide repeat protein with a globular fold. *FEBS J.*, **272**, 166–179.

12. Su, M.Y., Som, N., Wu, C.Y., Su, S.C., Kuo, Y.T., Ke, L.C., Ho, M.R., Tzeng, S.R., Teng, C.H., Mengin-Lecreulx, D., et al. (2017) Structural basis of adaptor-mediated protein degradation by the tail-specific PDZ-protease Prc. *Nat. Commun.*, **8**, 1–13.

13. Parashar, V., Aggarwal, C., Federle, M.J. and Neiditch, M.B. (2015) Rgg protein structure-function and inhibition by cyclic peptide compounds. *Proc. Natl. Acad. Sci. U. S. A.*, **112**, 5177–5182.

14. Wilkening, R. V, Capodagli, G.C., Khataokar, A., Tylor, K.M., Neiditch, M.B. and Federle, M.J. (2017) Activating mutations in quorum-sensing regulator Rgg2 and its conformational flexibility in the absence of an intermolecular disulfide bond. *J. Biol. Chem.*, **292**, 20544–20557.

15. Takayanagi, H., Yuzawa, S. and Sumimoto, H. (2015) Structural basis for the recognition of the scaffold protein Frmpd4/Preso1 by the TPR domain of the adaptor protein LGN. *Acta Crystallogr. Sect. F, Struct. Biol. Commun.*, **71**, 175–183.

16. Carminati, M., Gallini, S., Pirovano, L., Alfieri, A., Bisi, S. and Mapelli, M. (2016) Concomitant binding of Afadin to LGN and F-actin directs planar spindle orientation. *Nat. Struct. Mol. Biol.*, **23**, 155–163.

17. Yuzawa, S., Kamakura, S., Iwakiri, Y., Hayase, J. and Sumimoto, H. (2011) Structural basis for interaction between the conserved cell polarity proteins inscuteable and Leu-Gly-Asn repeat-enriched protein (LGN). *Proc. Natl. Acad. Sci. U. S. A.*, **108**, 19210–19215.

18. Pan, Z., Shang, Y., Jia, M., Zhang, L., Xia, C., Zhang, M., Wang, W. and Wen, W. (2013) Structural and biochemical characterization of the interaction between LGN and Frmpd1. *J. Mol. Biol.*, **425**, 1039–1049.

19. Zhu, J., Wen, W., Zheng, Z., Shang, Y., Wei, Z., Xiao, Z., Pan, Z., Du, Q., Wang, W. and Zhang, M. (2011) LGN/mInsc and LGN/NuMA complex structures suggest distinct functions in asymmetric cell division for the Par3/mInsc/LGN and Gαi/LGN/NuMA pathways. *Mol. Cell*, **43**, 418–431.

20. Wang, J., Dye, B.T., Rajashankar, K.R., Kurinov, I. and Schulman, B.A. (2009) Insights into anaphase promoting complex TPR subdomain assembly from a CDC26-APC6 structure. *Nat. Struct. Mol. Biol.*, **16**, 987–989.

21. Steegborn, C.S., Danot, O., Huber, R. and Clausen, T. (2001) Crystal structure of transcription factor MalT domain III: A novel helix repeat fold implicated in regulated oligomerization. *Structure*, **9**, 1051–1060.

22. Lee, C. and Goldberg, J. (2010) Structure of Coatomer Cage Proteins and the Relationship among COPI, COPII, and Clathrin Vesicle Coats. *Cell*, **142**, 123–132.

23. Dodonova, S.O., Diestelkoetter-Bachert, P., Von Appen, A., Hagen, W.J.H., Beck, R., Beck, M., Wieland, F. and Briggs, J.A.G. (2015) A structure of the COPI coat and the role of coat proteins in membrane vesicle assembly. *Science (80-.).*, **349**, 195–198.

24. Stanley, W.A., Filipp, F. V., Kursula, P., Schüller, N., Erdmann, R., Schliebs, W., Sattler, M. and Wilmanns, M. (2006) Recognition of a Functional Peroxisome Type 1 Target by the Dynamic Import Receptor Pex5p. *Mol. Cell*, **24**, 653–663.

25. Stanley, W.A., Pursiainen, N. V., Garman, E.F., Juffer, A.H., Wilmanns, M. and Kursula, P. (2007) A previously unobserved conformation for the human Pex5p receptor suggests roles for intrinsic flexibility and rigid domain motions in ligand binding. *BMC Struct. Biol.*, **7**, 1–12.

26. Sampathkumar, P., Roach, C., Michels, P.A.M. and Hol, W.G.J. (2008) Structural Insights into the Recognition of Peroxisomal Targeting Signal 1 by Trypanosoma brucei Peroxin 5. *J. Mol. Biol.*, **381**, 867–880.

Suppl. Table 3. RMSDs calculated from the superpositions of the full-length, the N-terminal domain and the C-terminal domains for all monomers in the apo and peptide-bound Rap crystal structures. The residue ranges are indicated, superpositions were done on the main chain atoms.

	Residues	6T3H A	6T3H B	6T46 A	6T46 C	6T46 E	6T46 G
6T3H B	9 - 361	1.386	NA				
	80 - 361	0.743					
	9 – 68	0.981					
	268 - 361	0.380					
6T46 A	9- 361	2.072	1.454	NA			
	80 – 361	1.138	0.951				
	9 – 68	0.778	1.034				
	268 - 361	0.505	0.563				
6T46 C	9 – 361	1.601	1.776	1.719	NA		
	80 – 361	1.236	1.186	0.583			
	9 – 68	0.957	1.500	1.072			
	268 - 361	0.572	0.649	0.435			
6T46 E	9 – 361	2.354	1.787	1.328	2.059	NA	
	80 – 361	1.578	1.357	0.914	1.119		
	9 – 68	1.025	1.496	1.073	0.792		
	268 - 361	0.607	0.696	0.490	0.530		
6T46 G	9 – 361	3.540	3.275	2.742	2.484	3.146	NA
	80 – 361	2.855	2.682	2.068	1.935	2.251	
	9 – 68	0.969	1.547	1.199	0.639	0.832	
	268 - 361	0.714	0.740	0.607	0.667	0.674	

Suppl. Table 4. RMSDs calculated for the N-terminal domains after superposition of residues 80-361 of the C-terminal domains for all monomers in the apo and peptide-bound Rap crystal structures. Superpositions were done on the main chain atoms.

	6T3H A	6T3H B	6T46 A	6T46 C	6T46 E	6T46 G
6T3H B	2.011	-				
6T46 A	2.980	1.288	-			
6T46 C	1.317	3.938	4.016	-		
6T46 E	2.295	2.001	1.726	4.384	-	
6T46 G	5.744	6.047	5.944	4.554	6.989	-

Suppl. Table 5. Estimation of the molecular weights from the elution volumes of the SEC experiments on the different mixture of pLS20 Rap / Rco / Phr.

Protein/complex	V_{el} (ml)	M_w (kDa)	Estimated M_w based on V_{el} (kDa)	Estimated number of monomers
Rap_{pLS20}	1.78	44.43	101	2.3 (dimer)
Rco_{pLS20}	1.80	20.32	94.4	4.6 (tetramer)
Rap_{pLS20} + $phr*_{pLS20}$	1.65	45.1	157	3.5 (tetramer)
Rap_{pLS20}:Rco_{pLS20}	1.47 *	N.A. **	N.A. *	N.A. *
Rap_{pLS20}:Rco_{pLS20}+$phr*_{pLS20}$	1.64	N.A. **	162	Tetramer

* The elution volume was out of the calibration range

** The MW cannot be calculated since the stoichiometries are unknown

Suppl. Table 6. Calculated fits of the available X-ray structures and the experimental SAXS curves of Rap_{pLS20} in absence and presence of the peptide. The fits were calculated for mixtures of the monomer and C-terminally induced dimer of Rap (M-D), and for mixtures of the same dimer with foot-to-foot induced tetramer (D-T).

[Rap] (mg/ml)	Phr*	M-D/D-T	Chi-square	Dimer	Tetramer
5	-	M-D	74.11		
5	-	D-T	1.97	0.476±0.002	0.524±0.001
2.5	-	M-D	18.07		
2.5	-	D-T	1.09	0.594±0.003	0.406±0.002
0.5	-	M-D	1.39		
0.5	-	D-T	1.02	0.782±0.011	0.218±0.009
5	+	M-D	44.27		
5	+	D-T	7.82	0.012±0.005	0.988±0.004
2.5	+	M-D	45.08		
2.5	+	D-T	3.84	0.000±0.000	1.000±0.001
0.5	+	M-D	4.64		
0.5	+	D-T	0.72	0.255±0.011	0.745±0.009

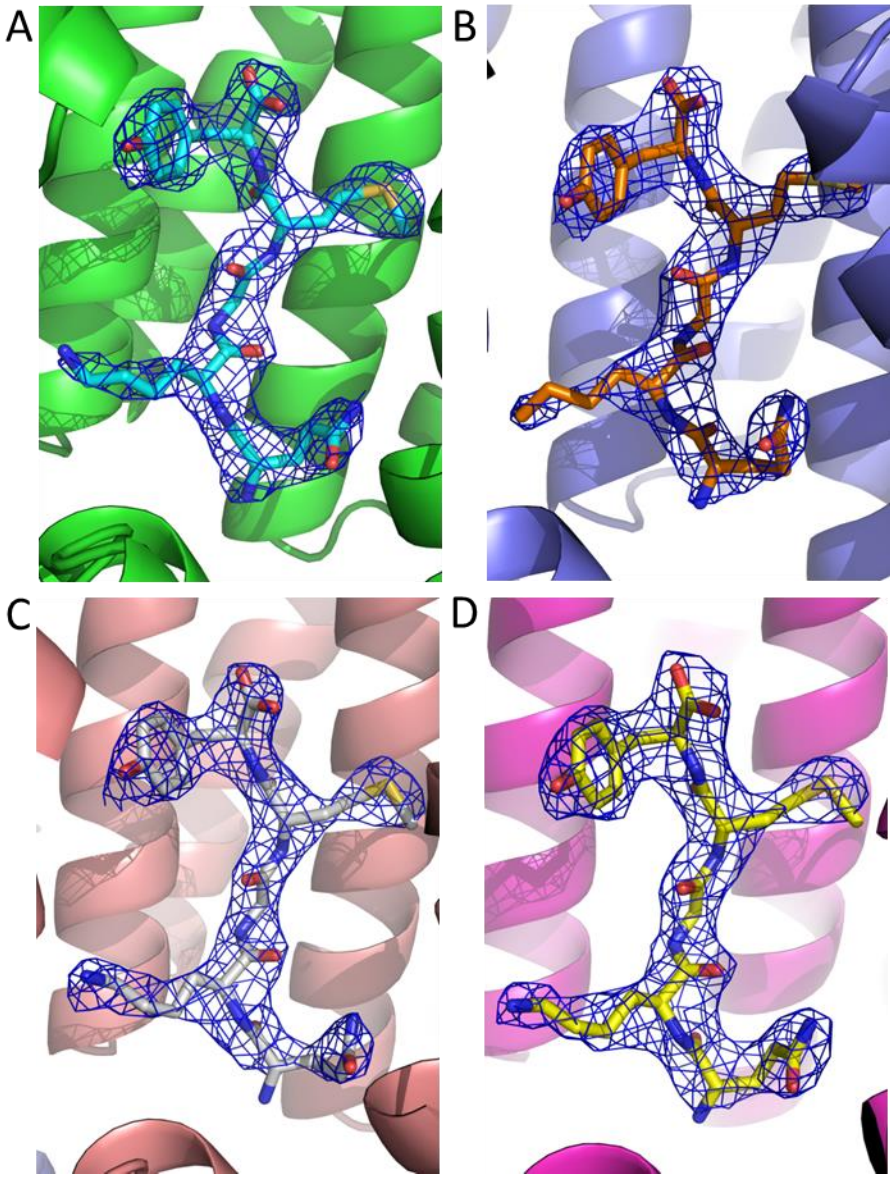

Suppl. Figure 1. Electron density maps around the peptide bound to Rap. Panels A) to D) show the peptide chains B, D, F and G of the Rap-peptide complex, respectively. The 2(mF_o-DF_c) density map is contoured at a 1.6 Å distance around the peptides and at a 1.2 σ cutoff.

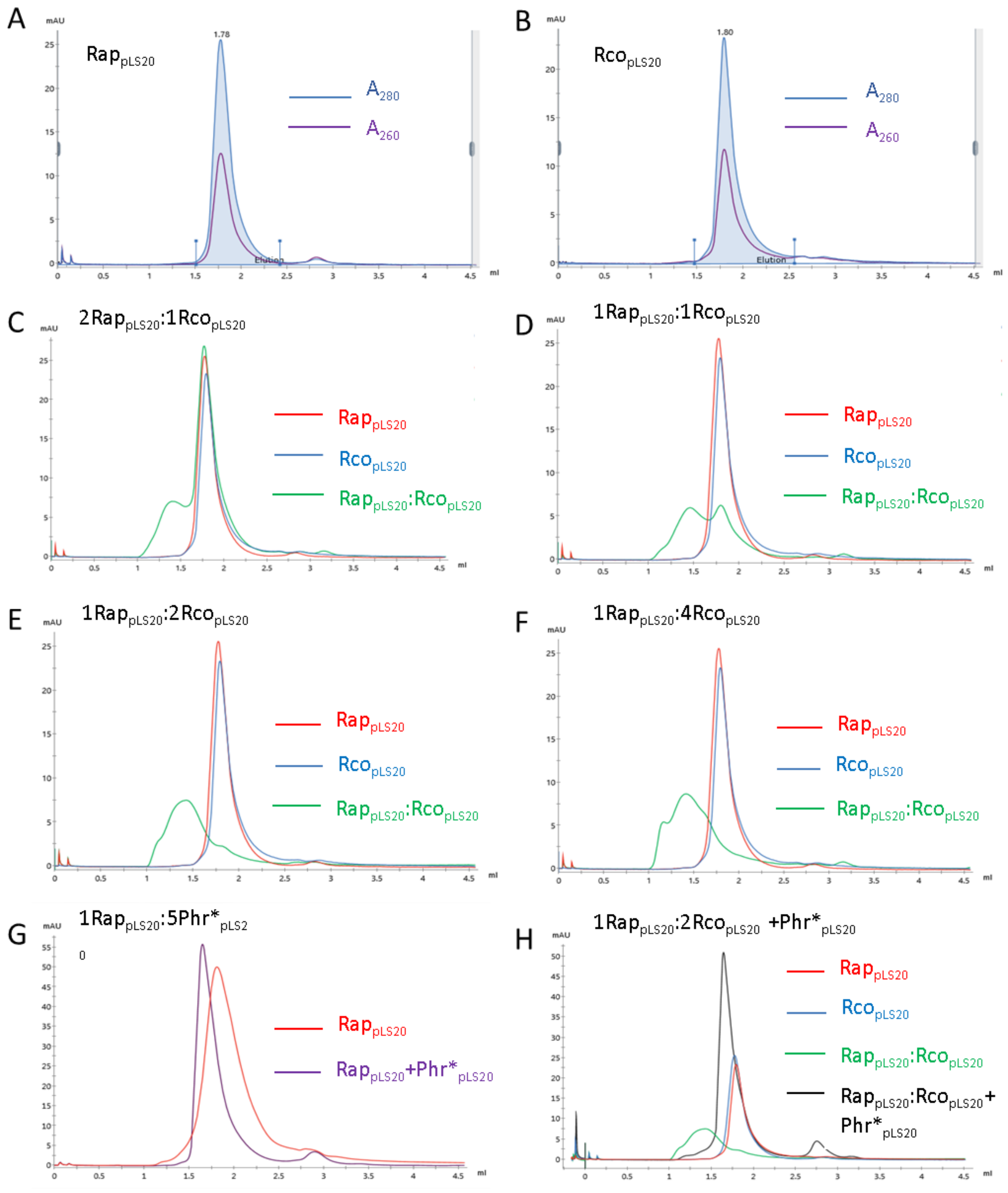

Suppl. Figure 2. Size Exclusion Experiments of Rap_{pLS20} (a), Rco_{pLS20} (b), Rap_{pLS20}:Rco_{LS20} complex at different molar ratios; 2:1 (c), 1:1 (d), 1:2(e) and 1:4 (f), Rap:Phr^{*} (g) and Phr^{*}_{pLS20} together with Rap_{pLS20}: Rco_{pLS20} complex (h). In a) and b) absorbance at 280 nm is represented in blue and absorbance at 260nm is represented in purple. In c), d), e), f) complex is represented in green, Rco_{pLS20} is shown in blue and Rap_{pLS20} in orange. In g) Rap_{pLS20}: Phr^{*}_{pLS20} corresponds to the green peak, Rap_{pLS20} to to the blue and

Phr^{*}_{pLS20} peptide to the orange. In h) the addition of Phr^{*}_{pLS20} to Rap_{pLS20}: Rco_{pLS20} complex is shown in purple, Rap_{pLS20}: Rco_{pLS20} complex in blue, apo Rap_{pLS20} in green and apo Rco_{pLS20} in orange.

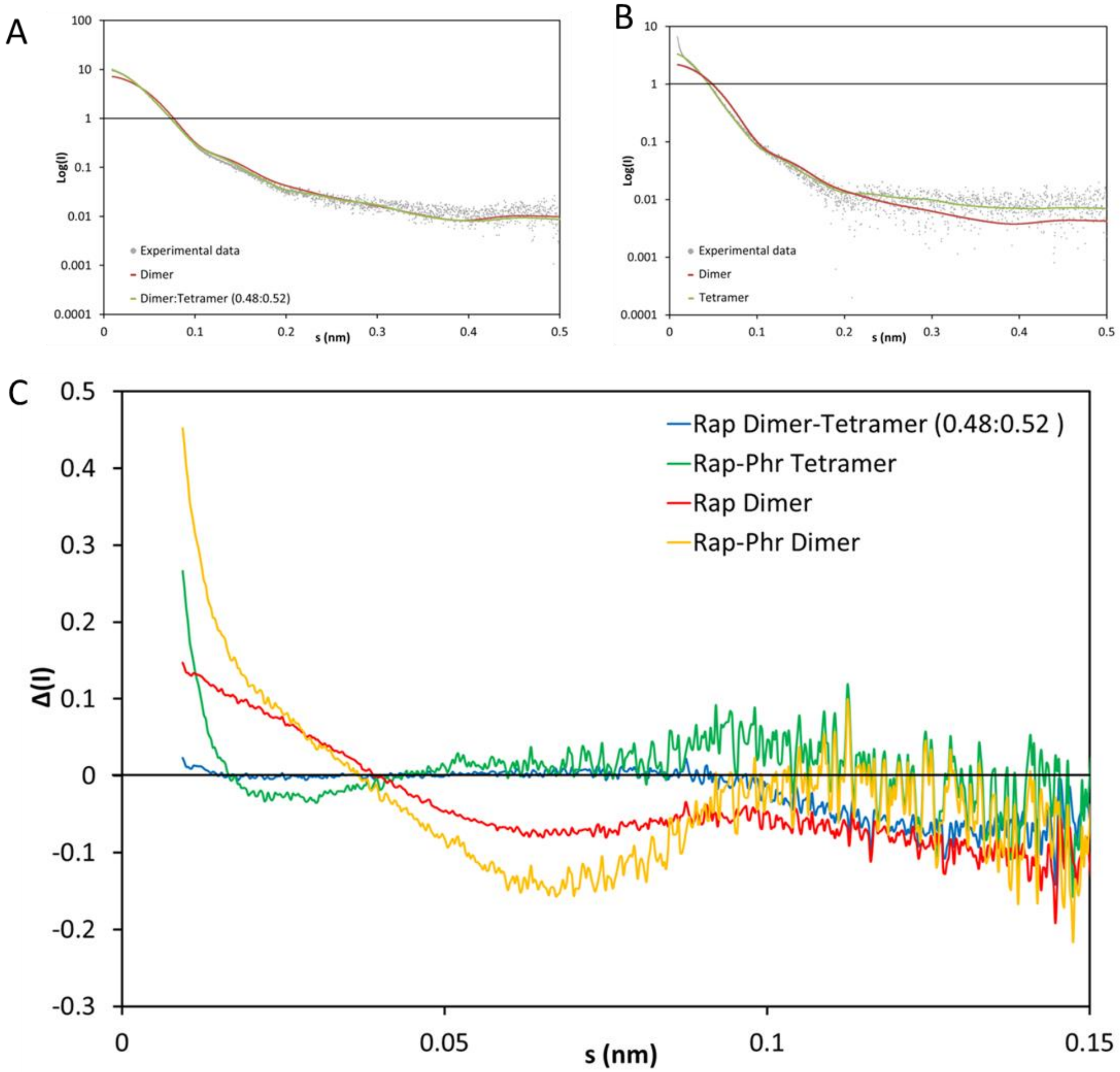

Suppl. Figure 3. A) Small-angle X-ray scattering curve of a solution of native Rap_{pLS20} at 5mg/ml. Experimental data are shown as gray dots, the dimer fit is shown in red and the fit of a dimer:tetramer mixture (0.48:0.52 stoichiometry) is shown in green. B) Small-angle X-ray scattering curve of a solution of Rap in complex with Phr^{*}_{pLS20} at 5mg/ml. Experimental data are shown as gray dots, the dimer fit is shown in red and the fit a tetramer is shown in green. C) Deviations of the fitted curves from experimental data in the 0–0.15 nm range. Blue: Rap 5mg/ml dimer-tetramer fit (0.48:0.52 stoichiometry), Red: Rap 5mg/ml dimer fit, Green: Rap-Phr* 5mg/ml tetramer fit and Orange: Rap-Phr* 5mg/ml dimer fit
